# A peptide derived from the N-terminus of charged multivesicular body protein 6 (CHMP6) promotes the secretion of gene editing proteins via small extracellular vesicle production

**DOI:** 10.1080/21655979.2022.2030571

**Published:** 2022-02-21

**Authors:** Junyu Fan, Jiajie Pan, Xiaozhe Zhang, Yixi Chen, Yue Zeng, Lihan Huang, Dongwei Ma, Ziqi Chen, Guifu Wu, Wendong Fan

**Affiliations:** a Department of Cardiology The Eighth Affiliated Hospital of Sun Yat-sen University Guangdong Shenzhen P.R. China; b Department of Cardiology, The First Affiliated Hospital of Sun Yat-sen University, Guangzhou, Guangdong, P.R. China; c NHC Key Laboratory of Assisted Circulation, Sun Yat-Sen University, Guangdong, Guangzhou, P.R. China; d Guangdong Innovative Engineering and Technology Research Center for Assisted Circulation Guangdong Shenzhen P.R. China

**Keywords:** Extracellular vesicles, genetic engineering, CHMP6, nanomedicine, Cre, Cas9

## Abstract

Extracellular vesicles (EVs) are a promising new therapeutic platform. However, the low cargo-loading efficiency limits their clinical translation. In this study, we developed a high-yield EV cargo-loading device and explored its ability to encapsulate gene editing proteins. A series of fusion protein-based systems were constructed and their cargo loading efficiencies were compared by a NanoGlo luciferase assay. A myristoylated (Myr) peptide tag cloned from the N-terminal region of charged multivesicular body protein 6 (CHMP6), termed Myr(CHMP6), outcompeted CD9, ARRDC1, and other short polypeptides as an active packaging device. As determined by nanoparticle tracking analysis and transmission electron microscopy, the overexpression of Myr(CHMP6) increased small EV (sEV) production in Lenti-X 293T  cells without altering sEV morphology. The high passive packaging efficiency of Myr(CHMP6) was also elucidated for unmodified cargo loading. Western blotting revealed that Myr(CHMP6) facilitated the loading of Cre and Cas9 into sEVs without the generation of packaging device-cargo fusion proteins. Furthermore, Myr(CHMP6)-modified sEVs loaded with Cre or Cas9 promoted gene-editing in recipient cells, as observed using a fluorescence reporter system. Subsequent investigation demonstrated a dose-dependent effect of Myr(CHMP6) tag-induced cargo-loading. Mechanistically, N-myristoylation alone was necessary but not sufficient for the effective packaging of proteins into EVs. Thus, our results indicated that Myr(CHMP6) induces sEV production and may be effective in loading gene editing proteins into sEVs for therapeutic purposes.

## Graphical Abstract

**Figure:**
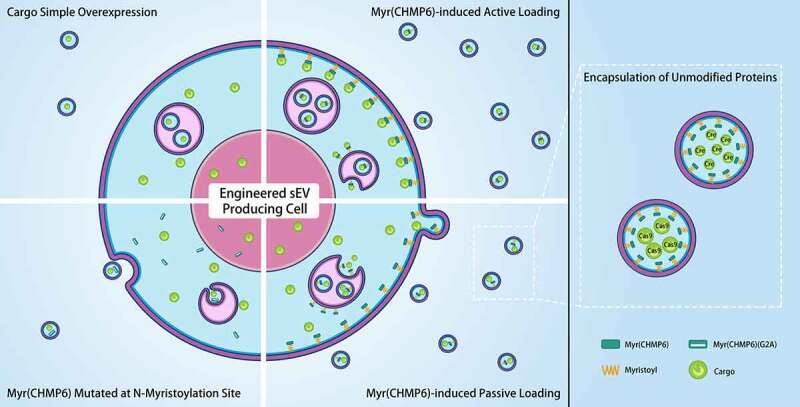

## 1. Introduction

Extracellular vesicles (EVs) are cell-derived vesicles capable of mediating intracellular communication. They are promising next-generation nanomedicines with intrinsic cargo-loading ability, nano-scale particle sizes, high biocompatibility, and targeting potential [1–3]. However, the limited productivity and cargo-loading efficiency of native EVs impede their application as a therapeutic platform.

EVs have recently been modified by re-engineering various EV-enriched proteins (EEPs), where EEPs are covalently connected to or selectively interact with cargoes, mediating their assembly into EVs. For example, an ARRDC1-p53 fusion structure could encapsulate p53 into EVs [4]. CD9 has also been fused to a blue light-controlled protein–protein interaction module for soluble protein loading [5]. Despite progress in the development of vesicle-mediated cargo delivery platforms, few studies have compared the cargo-loading efficiencies of different fusion protein-based methods, namely, active packaging devices [6–9]. Furthermore, active packaging requires design and manufacturing of new recombinant structures for each cargo of interest, limiting the practical application of engineered EVs.

Another widely used strategy for EV cargo loading is passive packaging. Cargoes are loaded into EVs in a dose-dependent, nonselective manner by overexpression. Despite relatively low yields, passive packaging remains the simplest method, as it does not require the design and construction of new recombinant proteins. This also allows us to encapsulate wild-type cargoes into EVs without fusing them to exogenous constructs, thus, avoiding changes in protein structure and subcellular distributions, and/or impaired cargo function caused by the fusion protein-based strategy.

Various post-translational modifications (PTMs) show regulatory effects on EV protein component sorting [10,11], prompting us to investigate whether polypeptides with PTMs can be re-engineered and used as an EV cargo-loading device. For example, myristoylation sites are found in essential components of viruses or endosomal sorting complexes required for transport (ESCRT), such as in the N-terminal arm of Tacaribe virus (TCRV) Z protein [12], human immunodeficiency virus (HIV) Gag protein [13], and ESCRT-III components CHMP3 [14] and CHMP6 [15]. Prenylation at C-terminal regions of the Rab GTPases is known to mediate their regulatory effect on vesicular transport [16–19]. In this study, after evaluating various candidate proteins with PTMs, we developed a novel packaging device, a 12 amino acid-long polypeptide from the N-terminus of the CHMP6 protein, termed Myr(CHMP6). We aimed to investigate whether Myr(CHMP6) is more efficient than traditional packaging methods using CD9- or ARRDC1- fusion proteins, and to explore its ability in delivering functional gene-editing proteins.

## 2. Materials and methods

### 2.1. Cell culture

Lenti-X 293T cells (632,180; Clontech, Mountain View, CA, USA) were maintained in Dulbecco’s modified Eagle medium (DMEM) with high glucose (HyClone, Logan, UT, USA), 10% fetal bovine serum (FBS; Gibco, Waltham, MA, USA), 100 units/mL penicillin G sodium, and 100 µg/mL streptomycin sulfate (Gibco).

### 2.2. Plasmids and transfection

Nluc-3×FLAG containing *Sal*I and *BsrG*I site was synthesized (Genewiz, Guangzhou, China) and inserted into pLV-mCherry (#36,084; Addgene, Watertown, MA, USA) using In-Fusion (Takara) to generate pLV-mCherry-Nluc-3×FLAG. Genes encoding CD9, ARRDC1, mono-ubiquitin (Ubi), and sonic hedgehog (SHH) were cloned from the  Lenti-X 293T cDNA library. Genes encoding polypeptides with myristoylation (Myr) or prenylation (Prenyl) sites were synthesized (Genewiz) or PCR-amplified from primer pairs complementary at the 3′ ends to generate primer dimers and then inserted at the 5′ end or 3′ end of mCherry-Nluc-3×FLAG with In-Fusion to generate recombinant plasmids encoding fusion protein-based cargo-loading systems. GFP was cloned from pGreenFire1-NF-kB (TR012PA-1; System Biosciences, Palo Alto, CA, USA) and the HA tag was synthesized (Genewiz). Overlap extension PCR was performed to generate GFP-HA, followed by In-Fusion insertion into pLV-mCherry (cut with *Sal*I and *BamH*I) to generate pLV-GFP-HA or into Myr(CHMP6)-mCherry-Nluc-3×FLAG (cut with *Sal*I and *Spe*I) to generate pLV-Myr(CHMP6)-GFP-HA. Cre-3×FLAG was synthesized and inserted into pLV-mCherry (cut with *Sal*I and *Xba*I) to generate pLV-Cre-3×FLAG. A G2A mutation was introduced in the Myr(CHMP6) tag using mutagenic primers. A fluorescence reporter system segment loxp-target-mCherry-stop-target-loxp-GFP-stop containing *BsmB*I and *Sal*I sites was synthesized (Genewiz) and inserted into pLV-mCherry with T4 DNA ligase (Thermo Scientific, Waltham, MA, USA) to generate pLV-Cre/Cas9-reporter. A segment encoding a U6 promoter and a sgRNA, termed sg-1, was synthesized (Genewiz) and In-Fusion-inserted into pLV-Cre/Cas9-reporter (cut with *Cla*I) to generate pLV-Cre/Cas9-reporter-sg-1. Inserted DNA fragments were confirmed by sequencing. Cells were transfected using Lipo293 Transfection Reagent (Beyotime, Shanghai, China) at 60–80% confluency according to the manufacturer protocol. Primers used to construct plasmids are listed in Table S1.

### 2.3. Microscopy for cell imaging

Transfected cells were seeded on 35 mm confocal dishes (Biofil, Guangzhou, China), cultured overnight, and examined using a LSM 780 laser scanning microscope (ZEISS, Oberkochen, Germany). GFP was excited with a 488 nm laser while mCherry was excited with a 561 nm laser.

### 2.4. Luciferase activity assay

Cells were seeded into 24-well plates. Culture medium was changed 24 h after transfection and collected after another 24 h, followed by sequential centrifugation at 300 × *g* for 10 min, 2000 × *g* for 10 min, and 10,000 × *g* for 30 min at 4°C to remove large debris. The supernatant was loaded onto a white 96-well plate (Corning, NY, USA), and 50 μL of Nano-Glo Luciferase Assay Substrate (Promega, Madison, WI, USA) diluted 1:50 with the provided buffer was added to each reaction. Cells in 24-well plates were lysed with 300 μL of 1× Passive Lysis Buffer (Promega) per well and centrifuged at 12,000 × *g* for 10 min. Nluc activity was measured in 50 μL of cell lysate supernatants diluted 1:1000.

### 2.5. sEV isolation

The sEVs were isolated via differential centrifugation [20,21]. Cells in 150 mm culture plates were washed and the culture medium was changed to 25 mL of fresh FBS-free DMEM with high glucose (HyClone) 24 h after transfection. After another 24 h, the culture medium was collected and sequentially centrifuged at 300 × *g* for 10 min, 2000 × *g* for 10 min, and 10,000 × *g* for 30 min, followed by filtration with a 0.22 μm syringe filter (Merck Millipore, Billerica, MA, USA). sEVs were pelleted by ultracentrifugation at 34,100 rpm (~120,000 × *g*) for 70 min at 4°C in an Optima XE-100 ultracentrifuge (Beckman Coulter, Brea, CA, USA) with the type 70 Ti rotor. Pellets were washed with phosphate-buffered saline (PBS; HyClone), re-ultracentrifuged, and resuspended with PBS (HyClone) or RIPA buffer (Thermo Scientific, Waltham, MA, USA).

### 2.6. sEV characterization

sEVs isolated from 25 mL of the culture supernatant were resuspended in 175 μL of PBS. For a nanoparticle tracking analysis (NTA), sEVs were analyzed using the NanoSight NS300 (Malvern Panalytical, Malvern, UK). For sEV visualization, 5–10 μL of resuspended sEVs was stained with phosphotungstic acid (PTA) on a copper grid and examined under a transmission electron microscope (JEM-1200EX; JEOL, Tokyo, Japan).

### 2.7. Western blotting

Cells or sEVs were lysed on ice in RIPA buffer (Thermo Scientific) supplemented with protease and phosphatase inhibitors (Beyotime) and then cleared by centrifugation at 12,000 × *g* for 10 min. The cell lysates were quantified with a Pierce BCA protein assay kit (Thermo Scientific) according to the manufacturer protocol. The lysates were then resuspended in SDS-PAGE sample loading buffer (Beyotime). Cell lysates were loaded into SDS-PAGE gels based on equal protein amount (2 μg), while sEV lysates were loaded based on equal volume (20 μL) to reflect sEV contents from the same number of cells. Samples were separated by SDS-PAGE and transferred onto a PVDF membrane (Merck Millipore). Blots were probed with primary antibodies in Tris-buffered saline containing 0.025% Tween-20 (TBST; Asegene, Guangzhou, China) containing 5% bovine serum albumin (BSA; Thermo Scientific), followed by incubation with HRP-conjugated anti-rabbit antibody or anti-mouse antibody. Primary antibodies are listed in Table S2. Protein bands were visualized with an ECL detection reagent (Merck Millipore) according to the manufacturer protocol and captured with the Amersham Imager 600 (General Electric), and quantified using ImageJ (NIH, Bethesda, ML, USA).

### 2.8. Gene editing protein delivery assay

Donor cells were co-transfected with packaging device Myr(CHMP6)-GFP-HA or its control GFP-HA, along with cargo-encoding plasmid pLV-Cre-3×FLAG, LentiCRISPRv2 (#52,961; Addgene), or the control pLV-mCherry-Nluc-3×FLAG. sEVs in the culture medium were isolated as described in Section 2.5. Recipient cells were incubated with isolated sEVs for 12 h and then transiently transfected with pLV-Cre/Cas9-reporter-sg-1. After another 48 h, the transfected reporter cells were examined using a LSM 780 laser scanning microscope (ZEISS).

### 2.9. Statistical analysis

Results are expressed as means ± standard error of the mean (SEM) and analyzed using SPSS 25.0 (IBM, Armonk, NY, USA). Comparisons between two groups were performed by unpaired two-tailed Student’s *t*-tests. Comparisons among three or more groups were performed by one-way analysis of variance (ANOVA) and Bonferroni correction. Values *p* < 0.05 were considered statistically significant.

## 3. Results

In this study, we aimed to develop a high-yield EV cargo-loading device. The Myr(CHMP6)-Cargo fusion structure was found to have significantly higher cargo-loading efficiencies than those of traditional CD9- or ARRDC1-Cargo loading systems. By co-expression with Myr(CHMP6)-GFP-HA, cargoes can be effectively loaded into sEVs without fusing to the packaging device. Our data indicated that Myr(CHMP6) can facilitate Cre and Cas9 protein-loading into sEVs and promote gene-editing in the recipient cells, supporting further applications in EV-based therapy. Lastly, we demonstrated a dose-dependent effect and the essential role of a Gly^2^ myristoylation site in Myr(CHMP6)-mediated cargo loading.

### 3.1. Myr(CHMP6) outcompetes various protein tags as an active packaging device

**Figure 1.**
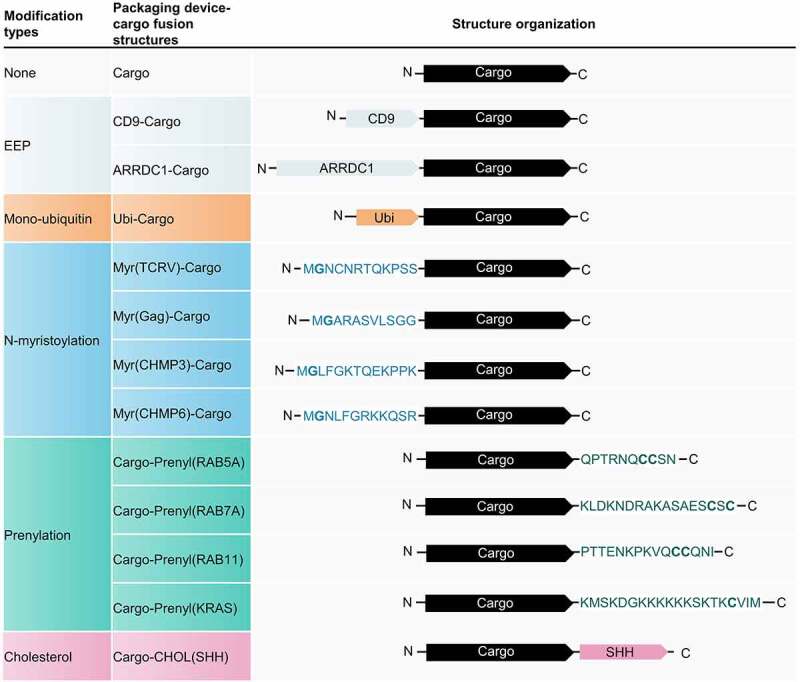
Schematic of different packaging device-cargo fusion structures. The cargo protein is composed of mCherry enabling live-cell imaging, a Nanoluciferase (Nluc) (which can be sensitively quantified by a NanoGlo luciferase assay), and a 3×FLAG tag capable of Western blot detection. Full-length CD9 or arrestin domain-containing protein 1 (ARRDC1) was fused to the N-terminus of the cargo as an EEP-Cargo strategy. A monoubiquitin was fused to the N-terminus of the cargo to investigate the packaging efficiency of mono-ubiquitination. The N-terminus of Tacaribe virus (TCRV) Z protein, HIV Gag protein, charged multivesicular body protein 3 (CHMP3), and charged multivesicular body protein 6 (CHMP6) were fused to the N-terminus of the cargo to assess the effect of N-myristoylation on cargo-loading. The C-termini of RAB5A, RAB7A, RAB11, and KRAS were fused to the C-terminus of the cargo to examine the effect of prenylation on cargo-loading. Sonic hedgehog (SHH) was also fused to the C-terminus of cargo to enable cholesterol attachment. EEP, Extracellular vesicle-enriched proteins.

To investigate whether polypeptides with PTMs can be re-engineered as EV cargo-loading devices, we cloned polypeptides from different genes containing potential PTM sites, including Ubi, Myr, Prenyl, or cholesterol (CHOL) attachment sites (Figure 1). Traditional active packaging methods, based on CD9 and ARRDC1, were used as positive controls. A cargo protein composed of mCherry, nanoluciferase (Nluc), and a 3×FLAG tag was designed for visualization and sensitive quantification.

**Figure 2.**
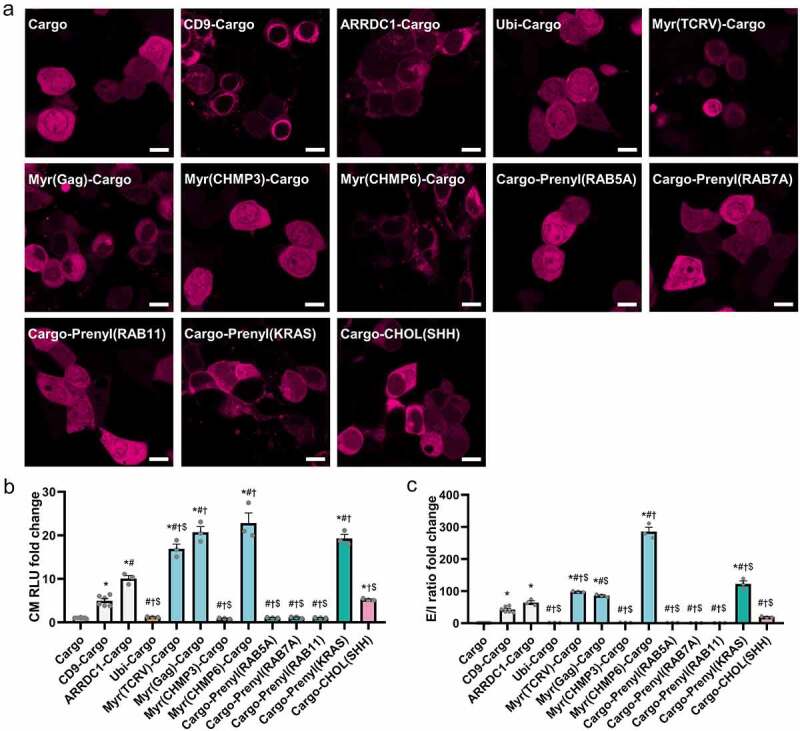
**Myr(CHMP6) outcompetes various protein tags as an active packaging device**. (a) Fluorescence images of cargoes fused to different packaging devices. Ubi-Cargo, Myr(CHMP3)-Cargo, Cargo-Prenyl(RAB5A), Cargo-Prenyl(RAB7A), and Cargo-Prenyl(RAB11) were uniformly distributed in cells, similar to the control group. Cargoes fused to CD9, ARRDC1, Myr(TCRV), Myr(Gag), Myr(CHMP6), Prenyl(KRAS), or CHOL(SHH) showed various altered distributions. (b) A NanoGlo luciferase assay was performed to evaluate different fusion protein-based active packaging systems. Nluc activity in the culture medium (CM) represents cargo release. Scale bar represents 10 μm. (c) The E/I ratio was calculated by dividing Nluc activity in the culture medium by Nluc activity in cell lysates (CL) of EV-producing cells. E/I ratio fold change represents fold change values of extracellular-to-intracellular RLU ratio. Bar graphs present fold change values compared to the first group as means ± standard error of the mean (SEM) (N ≥ 3). One-way ANOVA and Bonferroni correction were used for multiple comparisons to obtain adjusted *p*-values. *, Compared with Cargo group, Bonferroni adjusted *p* < 0.05; #, Compared with CD9-Cargo group, Bonferroni adjusted *p* < 0.05; †, Compared with ARRDC1-Cargo group, Bonferroni adjusted *p* < 0.05; $, Compared with Myr(CHMP6)-Cargo group, Bonferroni adjusted *p* < 0.05.

After transfection, Ubi-Cargo, Myr(CHMP3)-Cargo, Cargo-Prenyl(RAB5A), Cargo-Prenyl(RAB7A), and Cargo-Prenyl(RAB11) were uniformly distributed in cells, similar to the control (Figure 2(a) and Figure S1). Cargoes fused with CD9 were localized at the plasma membrane and in the perinuclear region. ARRDC1-Cargo, Myr(TCRV)-Cargo, Myr(Gag)-Cargo, Myr(CHMP6)-Cargo, Cargo-Prenyl(KRAS), and Cargo-CHOL(SHH) showed different levels of enrichment at the plasma membrane and punctate regions in the cytoplasm (Figure 2(a) and Figure S1).

A NanoGlo luciferase assay was then performed on culture medium (Figure 2(b)) and cell lysates (Figure S2). The extracellular-to-intracellular ratio (E/I ratio) was calculated by dividing Nluc activity in the culture medium by Nluc activity in cell lysates of EV-producing cells to investigate the cargo loading efficiency (Figure 2(c)). Fusion of CD9, ARRDC1, Myr(TCRV), Myr(GAG), Myr(CHMP6), Prenyl(KRAS), or CHOL(SHH) to the cargo protein induced a > 4-fold increase in cargo release and >40-fold increase in the E/I ratio. Myr(CHMP6) was the most effective active-packaging device (Figure 2(b,c)). After a proteinase K treatment on the culture medium, Myr(CHMP6)-Cargo still significantly increased cargo release and the E/I ratio (Figure S3). To further verify the EV-associated cargo loading, we examined the Nluc activities of CD63^+^ exosomes, which is an important subgroup of EVs. Myr(CHMP6)-Cargo induced a > 85-fold increase in cargo release in CD63^+^ exosomes purified by CD63 capture beads. And a > 578-fold increase in the exosome/cell cargo ratio. This result demonstrates that Myr(CHMP6) can promote cargo loading into CD63^+^ exosomes (Figure S4).

### 3.2. Myr(CHMP6) promotes sEV production

**Figure 3.**
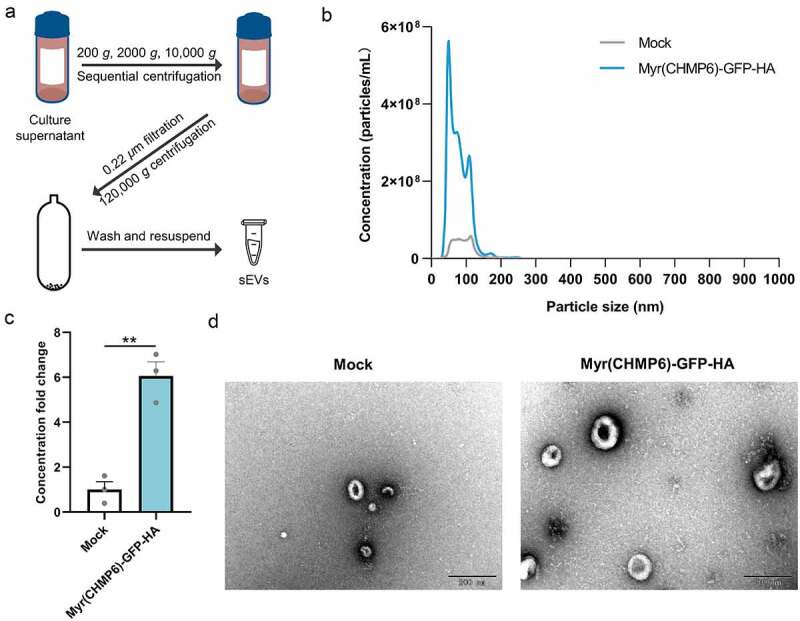
**Myr(CHMP6) boosts small extracellular vesicle (sEV) production**. (a) Experimental workflow of differential centrifugation to isolate sEVs. (b) Nanoparticle tracking analysis (NTA) of sEVs derived from Lenti-X 293T  cells transfected with the packaging device Myr(CHMP6)-GFP-HA or subjected to mock transfection. Data are presented as mean values of three biological replicates. (c) Quantitative sEV concentration from NTA. Data are presented as means ± SEM (N = 3). Statistical significance was assessed using unpaired two-tailed Student’s *t*-tests. **p < 0.01. (d) Representative electron microscopic images of sEVs. Scale bar represents 200 nm.

 Lenti-X 293T cells were transfected with Myr(CHMP6)-GFP-HA plasmids or subjected to mock transfection to determine whether the constructed packaging device affects sEV production. sEVs isolated by ultracentrifugation (Figure 3(a)) were examined by NTA, revealing that transfection with Myr(CHMP6)-GFP-HA increases sEV production (*p* = 0.002, Figure 3(b,c)). Furthermore, NTA and electron microscopy showed that the majority of Myr(CHMP6)-GFP-HA-induced sEVs were identical in size and morphology to those produced by mock control cells (Figure 3(b,d)). These results suggest that Myr(CHMP6) induces cargo protein secretion at least partially by promoting sEV production.

### 3.3. Myr(CHMP6) facilitated gene-editing protein loading into sEVs

**Figure 4.**
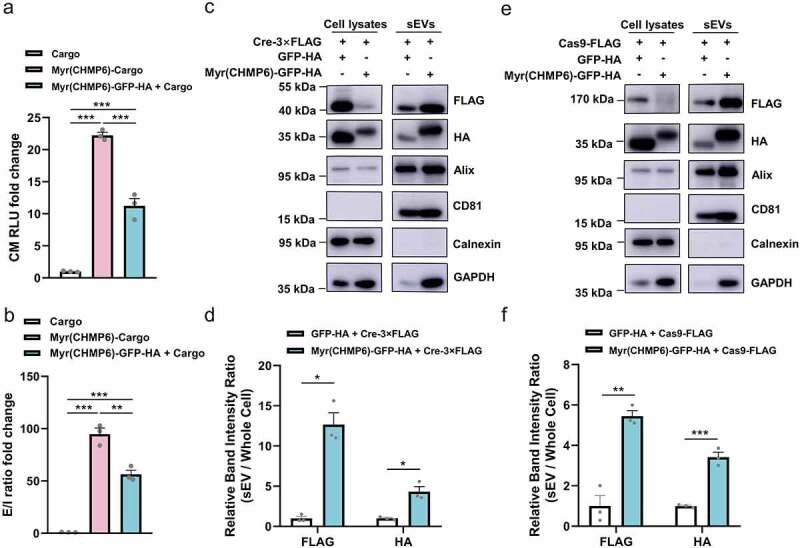
**Myr(CHMP6) facilitated gene-editing protein loading into sEVs**. (a) NanoGlo luciferase assay of Myr(CHMP6)-induced active and passive loading was performed. Nluc activity in the culture medium represents cargo release. (b) E/I ratio was calculated by dividing Nluc activity in the culture medium by Nluc activity in EV-producing cells. (c)  Lenti-X 293T cells were co-transfected with Cre-3×FLAG and Myr(CHMP6)-GFP-HA as indicated. Cell lysates and sEVs were collected and subjected to Western blotting with indicated antibodies. (d) Quantification of FLAG or HA enrichment in sEVs. FLAG or HA band intensity of sEVs was divided by the HA/Calnexin or FLAG/Calnexin intensity ratio for cell lysates. (e–f)  Lenti-X 293T cells were co-transfected with lentiCRISPR v2 (encoding Cas9-1×FLAG) and Myr(CHMP6)-GFP-HA, as indicated. Cell lysates and sEVs were collected and analyzed [as in (D)]. Data are presented as means ± SEM (N = 3). Unpaired two-tailed Student’s *t*-tests were used for comparisons between two groups. One-way ANOVA and Bonferroni correction were used for multiple comparisons to obtain adjusted *p*-values. *p < 0.05, **p < 0.01, ***p < 0.001.

We performed a NanoGlo luciferase assay to compare the efficiency of Myr(CHMP6)-mediated packaging of mCherry-Nluc-3×FLAG (the cargo protein) by active and passive loading (Figure 4(a,b), and Figure S5). The fusion protein Myr(CHMP6)-Cargo was used to examine the efficiency of active packaging, while co-expression of Myr(CHMP6)-GFP-HA and the cargo represents passive packaging. The Myr(CHMP6) co-expression strategy, though not as effective as the fusion-based method, significantly increased the productivity and E/I ratio of cargoes (all *p* < 0.01), indicating an effective passive cargo-loading device.

Exogenous cargoes for gene editing require nuclear transport, suggesting that passive loading may be an advantageous approach in gene therapy. To evaluate whether the co-expression of Myr(CHMP6)-GFP-HA can facilitate the packaging of proteins with nuclear localization signals (NLS), we further transfected  Lenti-X 293T cells with FLAG-tagged Cre or Cas9 with Myr(CHMP6)-GFP-HA. Western blotting showed enrichment of the exosomal markers Alix and CD81 but not the endoplasmic reticulum protein Calnexin in sEVs (Figure 4(c,e)), reflecting high-quality sEV isolation. An increase of Alix, CD81, and GAPDH (one of the top five most common proteins associated with EVs [22]) in sEVs was observed upon overexpression of Myr(CHMP6) (Figure 4(c,e)), supporting the increase of sEV production. Myr(CHMP6)-tagged GFP-HA was enriched in sEVs, along with co-transfected Cre or Cas9 (Figure 4(c-f)), supporting the nuclear protein packaging ability of Myr(CHMP6).)

### 3.4. Functionalized Myr(CHMP6)-sEVs enables efficient gene editing

**Figure 5.**
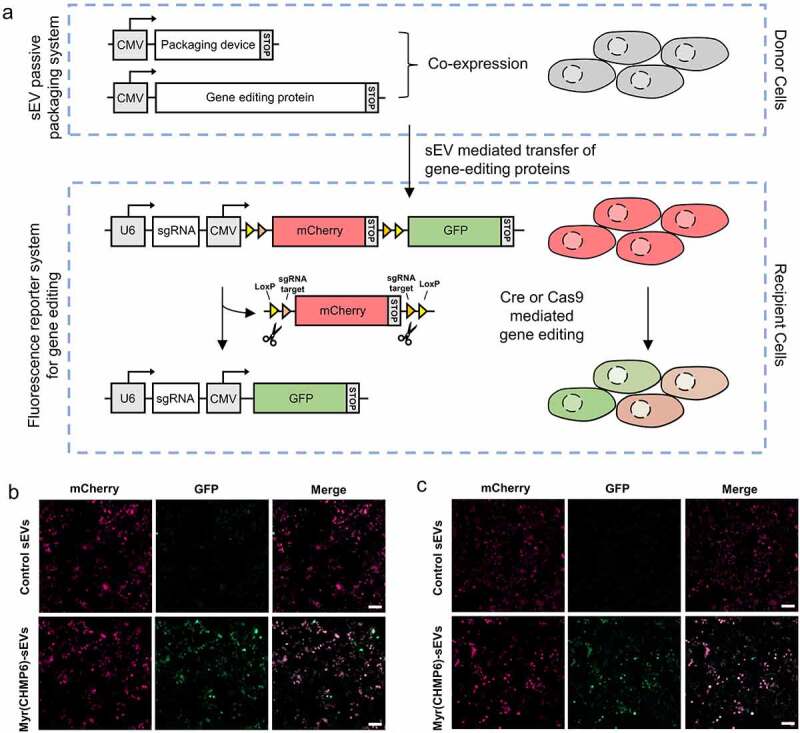
**Myr(CHMP6)-mediated protein delivery promotes gene editing**. (a) Schematic of the constructed system to report sEV mediated gene editing protein delivery from donor cells to recipient cells. The donor cells were transfected to co-express of a packaging device and gene-editing protein cargo. The recipient cells were treated with sEVs from donor cells, followed by transfection with a fluorescence reporter system plasmid pLV-Cre/Cas9-reporter-sg-1. The reporter system includes a U6 promoter which drives sgRNA transcription, and a CMV promoter which drives the transcription of sgRNA binding sites, LoxP and fluorescent proteins. The translation of the reporter system terminates after mCherry, inducing a red fluorescent signal. When functional Cre or Cas9 is delivered and induces knock out of mCherry and the first stop codon, translation stops after GFP, inducing a green fluorescent signal. (b) Representative fluorescent images of sEV-mediated gene editing. Recipient cells were treated with sEVs from donor cells expressing Myr(CHMP6)-GFP-HA (or GFP-HA) and Cre-3×FLAG, followed by transfection of the fluorescence reporter system. (c) Recipient cells were treated with sEVs from donor cells expressing Myr(CHMP6)-GFP-HA (or GFP-HA) and Cas9-FLAG, followed by transfection of the fluorescence reporter system. Myr(CHMP6)-sEVs, sEVs from donor cells that express Myr(CHMP6)-GFP-HA. Control sEVs, sEVs from donor cells that express GFP-HA. Scale bar represents 100 μm.

A fluorescence reporter system was constructed to explore whether the Myr(CHMP6)-engineered-sEVs promotes delivery of functional macro-molecular proteins for gene editing (Figure 5(a)). Donor cells were transfected with a packaging device (or a control plasmid) and a cargo protein. sEVs from donor cells that express packaging device Myr(CHMP6)-GFP-HA are termed Myr(CHMP6)-sEVs. sEVs from donor cells that express GFP-HA are termed control sEVs. Recipient cells were subjected to sEV treatment, followed by transfection of the fluorescence reporter system plasmid pLV-Cre/Cas9-reporter-sg-1. The gene editing events can be observed as the mCherry^+^GFP^−^ recipient cells would be recombined to mCherry^−^GFP^+^ cells (where all mCherry is knocked out) or mCherry^+^GFP^+^ cells (where a proportion of mCherry is knocked out) between indicated LoxP/sg-1 binding sites by bioactive Cre or Cas9. As confirmed by fluorescence microscopy, recipient cells treated with Myr(CHMP6)-sEVs loaded with Cre or Cas9 showed a red-to-green fluorescent shift (Figure 5(b,c)). In contrast, low GFP expression can be found in recipient cells treated with control sEVs loaded with Cre or Cas9 (Figure 5(b,c)). Myr(CHMP6)-sEVs or control sEVs without gene editing proteins are not able to induce the green fluorescent signal in recipient cells (Figure S6), precluding false positive result induced by the packaging device. These results demonstrate the functionality of gene editing proteins in recipient cells delivered by Myr(CHMP6)-sEVs.

### 3.5. Dose-dependent effect of Myr(CHMP6) tag on cargo loading

**Figure 6.**
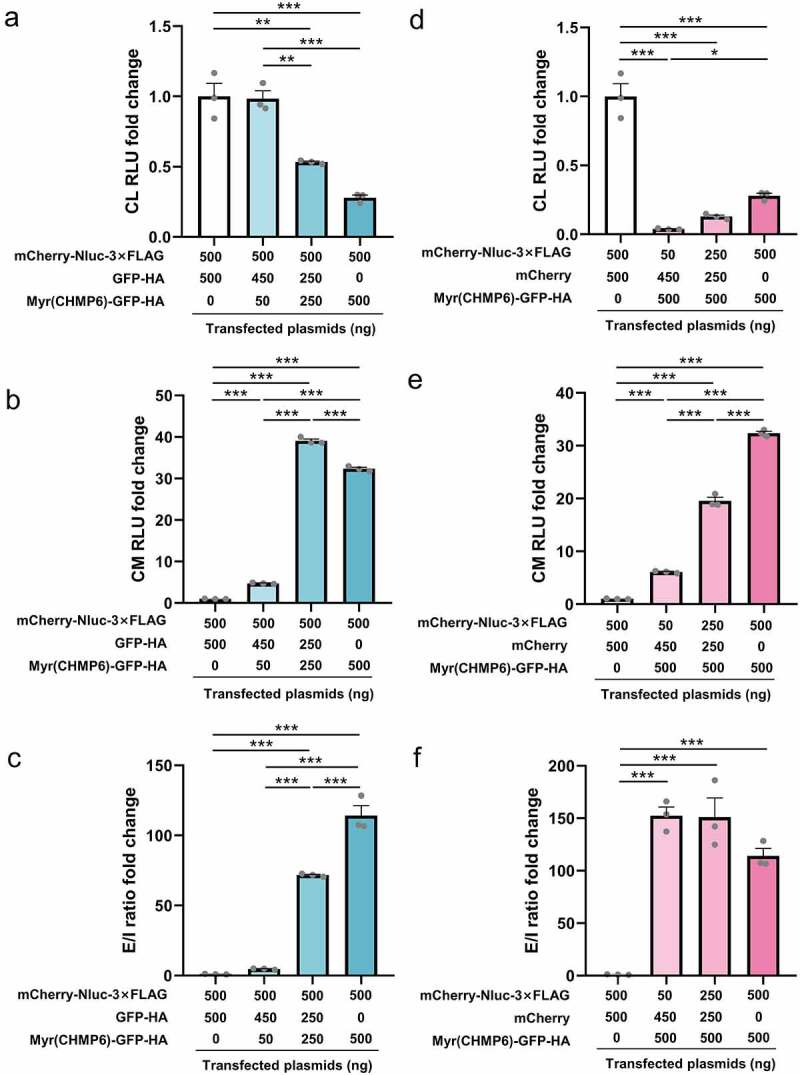
**Dose-dependent effect of Myr(CHMP6)-tag-induced cargo-loading**. Lenti-X 293T  cells in 24-well plates were transfected with cargo plasmids and the packaging device or control plasmids at the indicated doses. A NanoGlo luciferase assay was performed 48 h after transfection. (a-c) When cargo was fixed at 500 ng, Nluc activities in CL, culture medium (CM), and extracellular-to-intracellular RLU ratio (E/I ratio) were measured at different packaging device transfection doses. (d-f) With Myr(CHMP6)-GFP-HA fixed at 500 ng, Nluc activities in CL, CM, and E/L ratio were measured at different cargo transfection doses. Data are presented as means ± SEM (N = 3). One-way ANOVA and Bonferroni correction were used for multiple comparisons to obtain adjusted *p*-values. *p < 0.05, **p < 0.01, ***p < 0.001.

 Lenti-X 293T cells were transfected at different packaging device-to-cargo ratios to evaluate the relationship between Myr(CHMP6) expression levels and cargo protein transport. In this experiment, Myr(CHMP6)-GFP-HA was used as the passive packaging device for the cargo mCherry-Nluc-3×FLAG. Considering that the total plasmid amount may affect transfection efficiency, we used different doses of GFP-HA or mCherry to equalize the mass of Myr(CHMP6)-GFP-HA or mCherry-Nluc-3×FLAG, respectively. Interestingly, for an equal amount of cargo transfection, Nluc activity in cell lysates decreased as Myr(CHMP6) transfection increased (Figure 6(a)). The introduction of Myr(CHMP6) to the co-transfection system significantly increased cargo release (all *p* < 0.01, Figure 6(b)). The E/I ratio increased constantly with the increase in Myr(CHMP6) (Figure 6(c)). With equal doses of packaging device, cargo release increased with the level of cargo transfection (Figure 6(e)), while the E/I ratio did not significantly fluctuate (all *p* > 0.05, Figure 6(f)). These results demonstrated a dose-dependent effect of Myr(CHMP6)-tag-induced cargo loading.

### 3.6. The Gly^2^ myristoylation site is essential for the membrane affinity and packaging efficiency of Myr(CHMP6)

**Figure 7.**
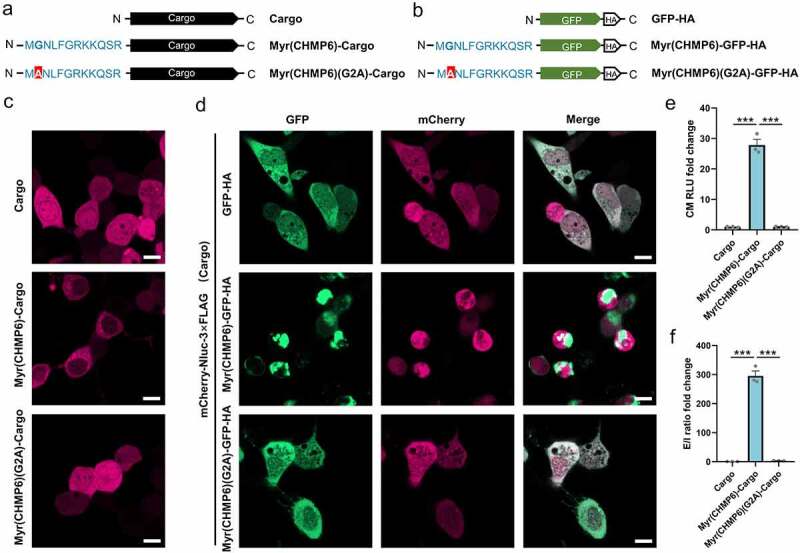
**The Gly^2^ myristoylation site is essential for the membrane affinity and packaging efficiency of Myr(CHMP6)**. (a) Schematic of the control cargo mCherry-Nluc-3×FLAG, active packaging device Myr(CHMP6)(G2A)-Cargo, and its mutant Myr(CHMP6)(G2A)-Cargo without the myristoylation site. (b) Schematic of GFP-HA, Myr(CHMP6)-GFP-HA, or the Gly^2^-mutated Myr(CHMP6)(G2A)-GFP-HA. (c) Representative fluorescence images of control cargo mCherry-Nluc-3×FLAG, active packaging device Myr(CHMP6)(G2A)-Cargo, and its mutant Myr(CHMP6)(G2A)-Cargo without the myristoylation site. Scale bar represents 10 μm. (d) Representative fluorescence images of cargo co-expressed with GFP-HA, Myr(CHMP6)-GFP-HA, or the mutated Myr(CHMP6)(G2A)-GFP-HA. Scale bar represents 10 μm. (e) NanoGlo luciferase assay of Myr(CHMP6) and its mutant Myr(CHMP6)(G2A)-induced active loading. Data are presented as means ± SEM (N = 3). One-way ANOVA and Bonferroni correction were used to obtain adjusted *p*-values for multiple comparisons. ***p < 0.001.

Myr(CHMP6)(G2A) mutants lacking the putative N-myristoylation site were generated to investigate the role of this modification (Figure 7(a,b)). While Myr(CHMP6)-Cargo distributed in the cytoplasm with enrichment at membrane structures (Figure 2(a), Figure 7(c) and Figure S1), the mutant Myr(CHMP6)(G2A)-Cargo was uniformly distributed in cells (Figure 7(c) and Figure S7), indicating that the G2A mutation decreased the membrane affinity of the Myr(CHMP6) tag. When co-transfected with the cargo, Myr(CHMP6)-GFP-HA showed a dot-like distribution in the cytoplasm and enrichment at the plasma membrane. The mCherry of the cargo remained generally freely distributed (Figure 7(d) and Figure S7). Interestingly, at sites with intense GFP signals, mCherry signals decreased and did not overlap with GFP, suggesting active packaging activity at the Myr(CHMP6)-modified locus (Figure 7(d) and Figure S7). In contrast, the localization of GFP-HA and the Myr(CHMP6)(G2A)-GFP-HA mutant was identical to that of the cargo (Figure 7(d) and Figure S7). Additionally, the G2A mutation abolished the ability of Myr(CHMP6) to induce cargo loading (Figure 7(e,f)). Taken together, these data demonstrated that Gly^2^ is essential for Myr(CHMP6)-induced cargo loading.

## 4. Discussion

We provide the first evidence that Myr(CHMP6), a short polypeptide tag, is an effective ‘EV production booster’ and can be used as both an active and passive cargo-loading device. As an active packaging device, the efficiency of Myr(CHMP6) outcompeted that of conventional methods utilizing CD9, ARRDC1, or other short protein tags. As a passive packaging device, the co-expression of Myr(CHMP6) enabled the encapsulation of unmodified cargoes, including Cre and Cas9, and induced gene editing in recipient cells. In light of the advantages of EV-based therapy, our packaging device may improve the cargo-loading ability and promote the clinical translation of engineered EVs.

Various methods, particularly active packaging, have been used to increase the cargo-loading efficiency of EVs. Constitutive proteins of EVs [4,5,23], virus-like particles [24,25], and other vesicle-enriched structures were re-engineered for direct or indirect interaction with modified cargoes. Ubiquitin is recognized by ESCRT [26,27]; however, our mono-ubiquitin tag did not substantially improve the packaging efficiency. Since EVs are largely enriched with cholesterol [28], we also cloned SHH for cholesterol attachment [29], which showed a high active packaging efficiency. The functions of many viral proteins, ESCRTIII components, and endosomal proteins are dependent on N-myristoylation [30–32] or prenylation [17]. Thus, we cloned the N- or C-terminus of EEPs with these lipidation sites as well as a prenylated KRAS, a truncated protein widely used as a membrane anchor [33], and fused these structures to the cargo (Figure 1). Some of these lipidation tags, namely Myr(TCRV), Myr(Gag), Myr(CHMP6), and Prenyl(KRAS), were more efficient than CD9 or ARRDC1 (Figure 2(b,c)). Although Myr(TCRV), Myr(Gag), Myr(CHMP3), and Myr(CHMP6) all included Gly^2^ myristoylation sites, their packaging efficiencies and membrane affinity differed (Figure 2). This observation demonstrates that downstream amino acid sequences, in addition to the myristoylation site itself, impact the cargo-loading efficiency of the packaging device. The Myr(CHMP6) tag was the most efficient active packaging device evaluated in this study (Figure 2(b,c)). Its packaging ability was further verified in proteinase K-treated culture medium and purified CD63^+^ exosomes (Figure S3 and Figure S4).

The majority of effective packaging devices interact with membranes. The tetraspanin CD9 has transmembrane domains. The arrestin-like domain of ARRDC1 interacts with the plasma membrane and regulates ARMM budding [34]. Lipidation tags regulate membrane trafficking and protein secretion [35]. In the present study, we found that few active-packaging structures remain in the nucleus (Figure 2(a)). This is an important limitation because therapeutic cargoes for gene therapy usually require nuclear transport. Further, the fusion of these devices inevitably changed the native sequence of cargoes. The packaging device itself as well as interacting molecules would attach to the cargo, potentially disrupting the functionality. For example, ARRDC1-p53 actively packaged in EVs attached to plasma membrane and was ubiquitinated [4], which might reduce the nuclear transport of p53 and preferentially lead to cargo degradation in target cells, thus impairing the overall bioactivity of the engineered EVs. Therefore, structural and functional validation of the cargoes are required for each new design, presenting an obstacle to the implementation of EV-based therapeutic technology. For the above reasons, simple overexpression is a common strategy for the loading of wild-type nuclear or cytosolic cargoes into EVs [36–38], which is rather low-yield, without increasing EV production [4,5,39]. To address these issues, we developed a simple but high-yield passive packaging strategy for unmodified wild-type cargoes. Since the Myr(CHMP6) tag is an efficient active-packaging device, we evaluated its utilization for passive-packaging. Transfection of Lenti-X 293T  cells with Myr(CHMP6)-GFP-HA significantly increased sEV production, without changing their morphology, demonstrating that it acts as an ‘EV production booster’ (Figure 3). Cargoes co-expressed with Myr(CHMP6) were effectively encapsuled (Figure 4(a,b)).

Cre/Loxp and CRISPR/Cas9 editing systems are unique technologies for genetic manipulation [40–42]. Many studies have attempted to encapsulate Cre or Cas9 into EVs by active loading. Cre, Cas9, or sgRNAs have been re-engineered to interact with EEPs or other EV-enriched structures via fusion with various modules [25,39,43–45]. Taking advantage of passive-loading, our Myr(CHMP6)-co-expression strategy enabled effective Cre and Cas9 encapsulation without cargo re-engineering (Figure 4). Notably, Myr(CHMP6) induced an increase in exosomal markers Alix and CD81, as well as GAPDH, which is reported to regulate EV biogenesis and binds to EV surface [46], supporting the ability of Myr(CHMP6) in promoting EV production. Furthermore, sEVs loaded with Cre or Cas9 were used to treat recipient cells expressing a fluorescence reporter system to induce gene editing. The gene-editing events in recipient cells were observed via fluorescence microscopy. When pre-treated with Myr(CHMP6)-sEVs, more recipient cells expressed GFP, indicating a higher efficiency of mCherry knock-out (Figure 5 and Figure S6). With these results, we demonstrate Myr(CHMP6) as an efficient tool to increase functional protein loading, even in the presence of NLS, into sEVs, with potential applications for gene therapy.

The dose-dependent effect was examined to investigate how Myr(CHMP6) affects protein trafficking. The results showed that the E/I ratio constantly increased with Myr(CHMP6) transfection, but not the amount of cargo transfected (Figure 6(c,f)), elucidating the role of Myr(CHMP6) in regulating cargo loading efficiency. In contrast, the amount of cargo released into the culture medium is related to transfection dose of both Myr(CHMP6) and the cargo (Figure 6(b,e)). Previous studies have provided evidence that N-myristoylation can target the AMP-activated protein kinase (AMPK) to lysosomes [47]. Our results showed a decrease of Nluc activity in cell lysates as well as the culture medium at the highest dose of Myr(CHMP6) transfection (Figure 6(a,b)). A possible explanation for this might be that the high dose of Myr(CHMP6) tag targeted cargo proteins to lysosomes and thus enhanced cargo degradation.

The Gly^2^ site of CHMP6 is crucial for its myristoylation, subcellular distribution and interaction with ESCRT components [15]. Our results showed that the G2A-mutated Myr(CHMP6) tag was uniformly distributed in the cytoplasm and nuclei, and it was not efficient in cargo loading (Figure 6 and Figure S7). Additionally, different peptide tags, although all contained N-myristoylation sites, had different packaging ability (Figure 2(b,c)), indicating that N-myristoylation alone was necessary but not sufficient for the effective packaging of proteins into EVs. Interestingly, we observed that cells transfected with Myr(CHMP6) are round and smaller than the control (Figure 6(d) and Fig S7). It was demonstrated that proteins with lipid affinity can regulate membrane shape by altering lipid bilayer force balance [48]. Since Myr(CHMP6)-tagged proteins showed high membrane affinity (Figure 2(a), Figure 7(c,d), Fig S1 and Fig S7), they might also be able to change the cell morphology.

Limitations of this study should also be noted. First, we did not evaluate the specificity of the Myr(CHMP6)-GFP-HA co-expression strategy. A mass spectrometry analysis of highly purified Myr(CHMP6)-induced EVs is necessary to characterize the changes in sEV composition and test the specificity of our system. Second, we did not address the mechanism by which Myr(CHMP6) induced sEV production. Previous studies have suggested that myristoylated proteins have high raft affinity [49,50] and that lipid raft components are involved in EV biogenesis [51]. Studies of the relationship between Myr(CHMP6) and lipid raft behavior may be helpful to understand the mechanism underlying elevated sEV production. Third, our data demonstrated the packaging ability and functionality of Myr(CHMP6)-induced sEVs with Cre or Cas9 proteins. Further *in vitro* and *in vivo* studies are needed to investigate the metabolic kinetics and targeting of these types of engineered EVs.

## 5. Conclusion

Myr(CHMP6) independently boosted sEV production and thus acted as an effective cargo-loading device. Myr(CHMP6) co-expression facilitated the encapsulation of Cre and Cas9 into sEVs, which further promoted gene editing in recipient cells. These finding indicates that Myr(CHMP6), as an effective packaging device, could be further used in gene therapy.

## Supplementary Material

Supplemental Material

## References

[1] Rani S, Ryan AE, Griffin MD, et al. Mesenchymal stem cell-derived extracellular vesicles: toward cell-free therapeutic applications. Mol Ther. 2015;23(5):812–823. 25868399 10.1038/mt.2015.44 PMC4427881

[2] Keshtkar S, Azarpira N, Ghahremani MH. Mesenchymal stem cell-derived extracellular vesicles: novel frontiers in regenerative medicine. Stem Cell Res Ther. 2018;9(1):63. 29523213 10.1186/s13287-018-0791-7 PMC5845209

[3] Hur YH, Cerione RA, Antonyak MA. Extracellular vesicles and their roles in stem cell biology. Stem Cells. 2020;38(4):469–476. 31828924 10.1002/stem.3140 PMC7703835

[4] Wang Q, Yu J, Kadungure T, et al. ARMMs as a versatile platform for intracellular delivery of macromolecules. Nat Commun. 2018;9(1):960. 29511190 10.1038/s41467-018-03390-x PMC5840177

[5] Yim N, Ryu SW, Choi K, et al. Exosome engineering for efficient intracellular delivery of soluble proteins using optically reversible protein-protein interaction module. Nat Commun. 2016;7:12277. 27447450 10.1038/ncomms12277 PMC4961865

[6] Silva AM, Lázaro-Ibáñez E, Gunnarsson A, et al. Quantification of protein cargo loading into engineered extracellular vesicles at single-vesicle and single-molecule resolution. J Extracell Vesicles. 2021;10(10):e12130. 34377376 10.1002/jev2.12130 PMC8329990

[7] Dooley K, McConnell RE, Xu K, et al. A versatile platform for generating engineered extracellular vesicles with defined therapeutic properties. Mol Ther. 2021;29(5):1729–1743. 33484965 10.1016/j.ymthe.2021.01.020 PMC8116569

[8] Gupta D, Liang X, Pavlova S, et al. Quantification of extracellular vesicles in vitro and in vivo using sensitive bioluminescence imaging. J Extracell Vesicles. 2020;9(1):1800222. 32944187 10.1080/20013078.2020.1800222 PMC7481830

[9] Corso G, Heusermann W, Trojer D, et al. Systematic characterization of extracellular vesicle sorting domains and quantification at the single molecule - single vesicle level by fluorescence correlation spectroscopy and single particle imaging. J Extracell Vesicles. 2019;8(1):1663043. 31579435 10.1080/20013078.2019.1663043 PMC6758720

[10] Moreno-Gonzalo O, Fernandez-Delgado I, Sanchez-Madrid F. Post-translational add-ons mark the path in exosomal protein sorting. Cell Mol Life Sci. 2018;75(1):1–19. 29080091 10.1007/s00018-017-2690-y PMC11105655

[11] Carnino JM, Ni K, Jin Y. Post-translational modification regulates formation and cargo-loading of extracellular vesicles. Front Immunol. 2020;11:948. 32528471 10.3389/fimmu.2020.00948 PMC7257894

[12] Fehling SK, Lennartz F, Strecker T. Multifunctional nature of the arenavirus RING finger protein Z. Viruses. 2012;4(11):2973–3011. 23202512 10.3390/v4112973 PMC3509680

[13] Bussienne C, Marquet R, Paillart JC, et al. Post-translational modifications of retroviral HIV-1 gag precursors: an overview of their biological role. Int J Mol Sci. 2021;22:6. 10.3390/ijms22062871 PMC8000049 33799890

[14] The UniProt Consortium . UniProt: the universal protein knowledgebase in 2021. Nucleic Acids Res. 2021;49(D1):D480–D489. 33237286 10.1093/nar/gkaa1100 PMC7778908

[15] Yorikawa C, Shibata H, Waguri S, et al. Human CHMP6, a myristoylated ESCRT-III protein, interacts directly with an ESCRT-II component EAP20 and regulates endosomal cargo sorting. Biochem J. 2005;387(Pt1):17–26. 10.1042/bj20041227. 15511219 PMC1134928

[16] Bhogaraju S, Taschner M, Morawetz M, et al. Crystal structure of the intraflagellar transport complex 25/27. Embo J. 2011;30(10):1907–1918. 21505417 10.1038/emboj.2011.110 PMC3098482

[17] Gomes AQ, Ali BR, Ramalho JS, et al. Membrane targeting of Rab GTPases is influenced by the prenylation motif. Mol Biol Cell. 2003;14(5):1882–1899. 12802062 10.1091/mbc.E02-10-0639 PMC165084

[18] Li F, Yi L, Zhao L, et al. The role of the hypervariable C-terminal domain in Rab GTPases membrane targeting. Proc Natl Acad Sci U S A. 2014;111(7):2572–2577. 24550285 10.1073/pnas.1313655111 PMC3932868

[19] Rab SH. GTPases as coordinators of vesicle traffic. Nat Rev Mol Cell Biol. 2009;10(8):513–525. 19603039 10.1038/nrm2728

[20] Coumans FAW, Brisson AR, Buzas EI, et al. Methodological guidelines to study extracellular vesicles. Circ Res. 2017;120(10):1632–1648. 28495994 10.1161/CIRCRESAHA.117.309417

[21] Yang D, Zhang W, Zhang H, et al. Progress, opportunity, and perspective on exosome isolation - efforts for efficient exosome-based theranostics. Theranostics. 2020;10(8):3684–3707. 32206116 10.7150/thno.41580 PMC7069071

[22] Kalra H, Simpson RJ, Ji H, et al. Vesiclepedia: a compendium for extracellular vesicles with continuous community annotation. PLoS Biol. 2012;10(12):e1001450. 23271954 10.1371/journal.pbio.1001450 PMC3525526

[23] Li Z, Zhou X, Wei M, et al. In Vitro and in Vivo RNA inhibition by CD9-HuR functionalized exosomes encapsulated with miRNA or CRISPR/dCas9. Nano Lett. 2019;19(1):19–28. 30517011 10.1021/acs.nanolett.8b02689

[24] Geeurickx E, Tulkens J, Dhondt B, et al. The generation and use of recombinant extracellular vesicles as biological reference material. Nat Commun. 2019;10(1):3288. 31337761 10.1038/s41467-019-11182-0 PMC6650486

[25] Zhang X, Xu Q, Zi Z, et al. Programmable extracellular vesicles for macromolecule delivery and genome modifications. Dev Cell. 2020;55(6):784–801 e9. 33296682 10.1016/j.devcel.2020.11.007 PMC9719439

[26] Kuo L, Freed EO. ARRDC1 as a mediator of microvesicle budding. Proc Natl Acad Sci U S A. 2012;109(11):4025–4026. 10.1073/pnas.1201441109 22378650 PMC3306698

[27] Chen Y, Zhao Y, Yin Y, et al. Mechanism of cargo sorting into small extracellular vesicles. Bioengineered. 2021;12(1):8186–8201. 34661500 10.1080/21655979.2021.1977767 PMC8806638

[28] Skotland T, Sandvig K, Llorente A. Lipids in exosomes: current knowledge and the way forward. Prog Lipid Res. 2017;66:30–41. 28342835 10.1016/j.plipres.2017.03.001

[29] Gallet A, Ruel RRL, Therond PP. Cholesterol modification of hedgehog is required for trafficking and movement, revealing an asymmetric cellular response to hedgehog. Dev Cell. 2003;4(2):191–204. 12586063 10.1016/s1534-5807(03)00031-5

[30] Morikawa Y, Hinata S, Tomoda H, et al. Complete inhibition of human immunodeficiency virus Gag myristoylation is necessary for inhibition of particle budding. J Biol Chem. 1996;271(5):2868–2873. 8576268 10.1074/jbc.271.5.2868

[31] Perez M, Greenwald DL. de la Torre JC. Myristoylation of the RING finger Z protein is essential for arenavirus budding. J Virol. 2004;78(20):11443–11448. 15452271 10.1128/JVI.78.20.11443-11448.2004 PMC521847

[32] Strecker T, Maisa A, Daffis S, et al. The role of myristoylation in the membrane association of the Lassa virus matrix protein Z. Virol J. 2006;3:93. 17083745 10.1186/1743-422X-3-93 PMC1647273

[33] Khan I, Rhett JM, O’Bryan JP. Therapeutic targeting of RAS: new hope for drugging the “undruggable”. Biochim Biophys Acta Mol Cell Res. 2020;1867(2):118570. 31678118 10.1016/j.bbamcr.2019.118570 PMC6937383

[34] JF N, Hu R, RS O, et al. Formation and release of arrestin domain-containing protein 1-mediated microvesicles (ARMMs) at plasma membrane by recruitment of TSG101 protein. Proc Natl Acad Sci U S A. 2012;109(11):4146–4151. 10.1073/pnas.1200448109 22315426 PMC3306724

[35] Jiang H, Zhang X, Chen X, et al. Protein lipidation: occurrence, mechanisms, biological functions, and enabling technologies. Chem Rev. 2018;118(3):919–988. 29292991 10.1021/acs.chemrev.6b00750 PMC5985209

[36] de Jong OG, DE M, Mäger I, et al. A CRISPR-Cas9-based reporter system for single-cell detection of extracellular vesicle-mediated functional transfer of RNA. Nat Commun. 2020;11(1):1113. 10.1038/s41467-020-14977-8 32111843 PMC7048928

[37] Chivero ET, Liao K, Niu F, et al. Engineered Extracellular vesicles loaded with miR-124 attenuate cocaine-mediated activation of microglia. Front Cell Dev Biol. 2020;8:573. 32850781 10.3389/fcell.2020.00573 PMC7409518

[38] Nie H, Jiang Z. Bone mesenchymal stem cell-derived extracellular vesicles deliver microRNA-23b to alleviate spinal cord injury by targeting toll-like receptor TLR4 and inhibiting NF-κB pathway activation. Bioengineered. 2021;12(1):8157–8172. 10.1080/21655979.2021.1977562 34663169 PMC8806461

[39] Sterzenbach U, Putz U, Low LH, et al. Engineered exosomes as vehicles for biologically active proteins. Mol Ther. 2017;25(6):1269–1278. 28412169 10.1016/j.ymthe.2017.03.030 PMC5474961

[40] Ekn G, Motta SE, Martin M, et al. Human iPSCs and genome editing technologies for precision cardiovascular tissue engineering. Front Cell Dev Biol. 2021;9:639699. 34262897 10.3389/fcell.2021.639699 PMC8273765

[41] Izumiya M, Kato S, Hippo Y. Recent advances in implantation-based genetic modeling of biliary carcinogenesis in mice. Cancers (Basel). 2021;13:10. 10.3390/cancers13102292 PMC8151177 34064809

[42] Zhao Z, Li C, Tong F, et al. Review of applications of CRISPR-Cas9 gene-editing technology in cancer research. Biol Proced Online. 2021;23(1):14. 10.1186/s12575-021-00151-x. 34261433 PMC8281662

[43] Heath N, Osteikoetxea X, de Oliveria TM, et al. Endosomal escape enhancing compounds facilitate functional delivery of extracellular vesicle cargo. Nanomedicine (Lond). 2019;14(21):2799–2814. 10.2217/nnm-2019-0061. 31724479

[44] Ye Y, Zhang X, Xie F, et al. An engineered exosome for delivering sgRNA:Cas9 ribonucleoprotein complex and genome editing in recipient cells. Biomater Sci. 2020;8(10):2966–2976. 32342086 10.1039/d0bm00427h

[45] Yao X, Lyu P, Yoo K, et al. Engineered extracellular vesicles as versatile ribonucleoprotein delivery vehicles for efficient and safe CRISPR genome editing. J Extracell Vesicles. 2021;10(5):e12076. 33747370 10.1002/jev2.12076 PMC7962171

[46] Dar GH, Mendes CC, Kuan WL, et al. GAPDH controls extracellular vesicle biogenesis and enhances the therapeutic potential of EV mediated siRNA delivery to the brain. Nat Commun. 2021;12(1):6666. 34795295 10.1038/s41467-021-27056-3 PMC8602309

[47] Garcia D, Shaw RJAMPK. Mechanisms of cellular energy sensing and restoration of metabolic balance. Mol Cell. 2017;66(6):789–800. 28622524 10.1016/j.molcel.2017.05.032 PMC5553560

[48] Frolov VA, Shnyrova AV, Zimmerberg J. Lipid polymorphisms and membrane shape. Cold Spring Harb Perspect Biol. 2011;3(11):a004747. 21646378 10.1101/cshperspect.a004747 PMC3220359

[49] Li SP, Lin ZX, Jiang XY, et al. Exosomal cargo-loading and synthetic exosome-mimics as potential therapeutic tools. Acta Pharmacol Sin. 2018;39(4):542–551. 29417947 10.1038/aps.2017.178 PMC5888690

[50] van Niel G, D’Angelo G, Raposo G. Shedding light on the cell biology of extracellular vesicles. Nat Rev Mol Cell Biol. 2018;19(4):213–228. 29339798 10.1038/nrm.2017.125

[51] Skryabin GO, Komelkov AV, Savelyeva EE, et al. Lipid Rafts in Exosome Biogenesis. Biochemistry (Mosc). 2020; 85(2):177–91. 10.1134/s0006297920020054. 32093594

