# Supplementary material for: A peptide derived from the N-terminus of charged multivesicular body protein 6 (CHMP6) promotes the secretion of gene editing proteins via small extracellular vesicle production
Source: Bioengineered. 2022 Feb 21;13(3):4702–16. doi: 10.1080/21655979.2022.2030571 (PMC8973635; doi:10.1080/21655979.2022.2030571)
Supplement: Supplemental Material [file KBIE_A_2030571_SM1069.zip › supplementary/Full_Supplementary Materials_revised.docx]

*Supplementary materials*

**A peptide derived from the N-terminus of charged multivesicular body protein 6 (CHMP6) promotes the secretion of gene editing proteins via small extracellular vesicle production**

Junyu Fan^1, 3, †^, Jiajie Pan^1, 3, †^, Xiaozhe Zhang^1, 3^, Yixi Chen^1, 3^, Yue Zeng^1, 3^, Lihan Huang^1, 3^, Dongwei Ma^1, 3^, Ziqi Chen^1, 3^, Guifu Wu^1, 3, 4, *^, and Wendong Fan^2, 3, *^

^1^Department of Cardiology, the Eighth Affiliated Hospital of Sun Yat-sen University, Shenzhen, 518033, Guangdong, P. R. China

^2^Department of Cardiology, the First Affiliated Hospital of Sun Yat-sen University, Guangzhou, 510080, Guangdong, P.R. China

^3^NHC Key Laboratory of Assisted Circulation (Sun Yat-sen University)

^4^Guangdong Innovative Engineering and Technology Research Center for Assisted Circulation

*Correspondence:

Wendong Fan, Department of Cardiology, the First Affiliated Hospital of Sun Yat-sen University, No. 58 Zhongshan 2nd Road, Guangzhou 510080, Guangdong, P.R. China, Tel: +86 020-87330396, Fax: +86 020-87330396, E-mail: [fanwd3@mail.sysu.edu.cn](mailto:fanwd3@mail.sysu.edu.cn) ;

Guifu Wu, Department of Cardiology, the Eighth Affiliated Hospital of Sun Yat-sen University, No. 3025 Shennan Zhong Road, Shenzhen 518033, Guangdong, P.R. China, Tel: +86 0755-83982222, Fax: +86 0755-83980805, E-mail: [wuguifu@mail.sysu.edu.cn](mailto:wuguifu@mail.sysu.edu.cn)

^†^ These authors contributed equally.

Supplementary methods

Proteinase K treatment

Cells were seeded on 6-well plates. Culture medium was changed 24 h after transfection and collected after another 24 h, followed by sequential centrifugation to remove large debris. The supernatants were treated with 10 μg/mL proteinase K (Beyotime) at 37℃ for 1h, and then inhibited with 5 mM PMSF (Beyotime) on ice for 5 min.

CD63^+^ exosome analysis

Culture mediums were collected and subjected to sequential centrifugation. 200 μL supernatant was incubated with 50 μL CD63 exosome capture beads (Abcam, Cambridge, MA, USA) at room temperature overnight. The exosome-loaded beads were then washed with ice-cold PBS, resuspended with 50 μL 1× Passive Lysis Buffer (Promega) and subjected to luciferase activity assay.

Supplementary Figures


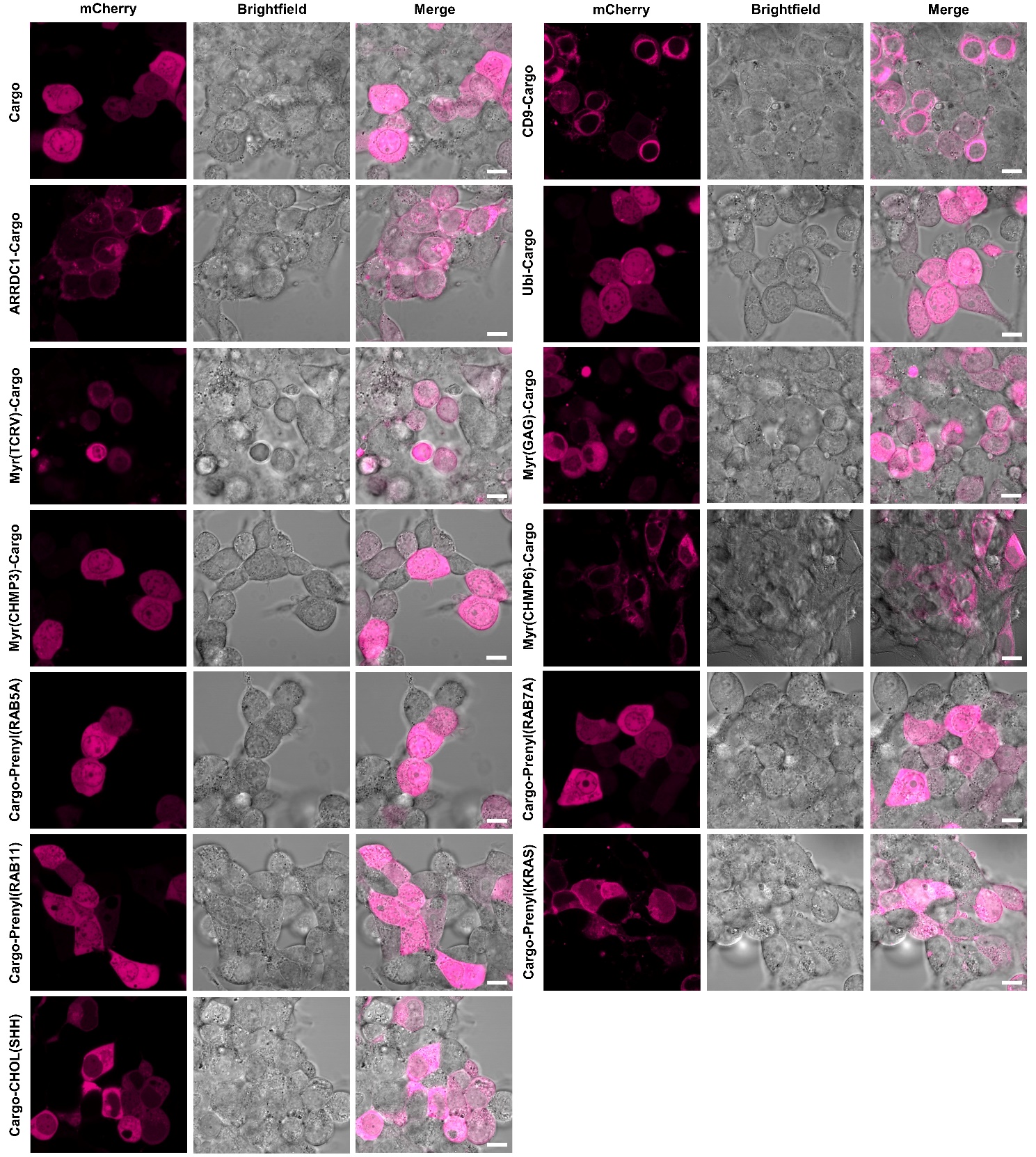


**Figure S1. Fluorescence and bright field images of constructed active-packaging devices.** Scale bar represents 10 μm.

**
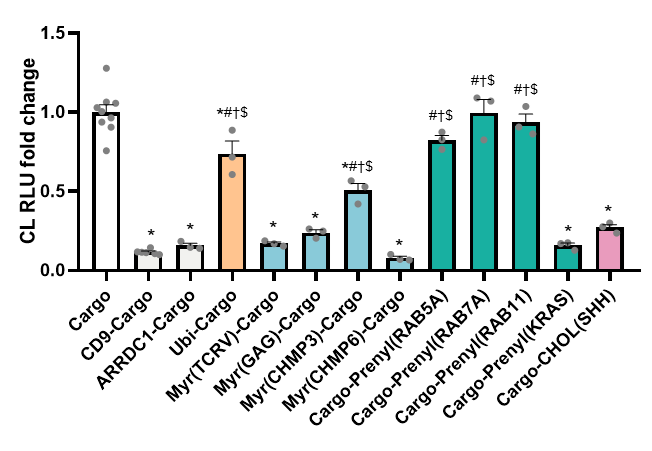
**

**Figure S2.** **Nluc activity of cell lysates (CL) corresponding to Figure 2B and 2C.** LentiX-293T cells in 24-well plates were transfected with chimeric cargo with or without fusion to active packaging devices. Bar graphs present fold change values compared to the first group as means ± standard error of the mean (SEM) (N ≥ 3). One-way ANOVA and Bonferroni correction were used for multiple comparisons to obtain adjusted *p*-values. *, Compared with Cargo group, Bonferroni adjusted *p* <0.05; #, Compared with CD9-Cargo group, Bonferroni adjusted *p* <0.05; †, Compared with ARRDC1-Cargo group, Bonferroni adjusted *p* <0.05; $, Compared with Myr(CHMP6)-Cargo group, Bonferroni adjusted *p* <0.05.


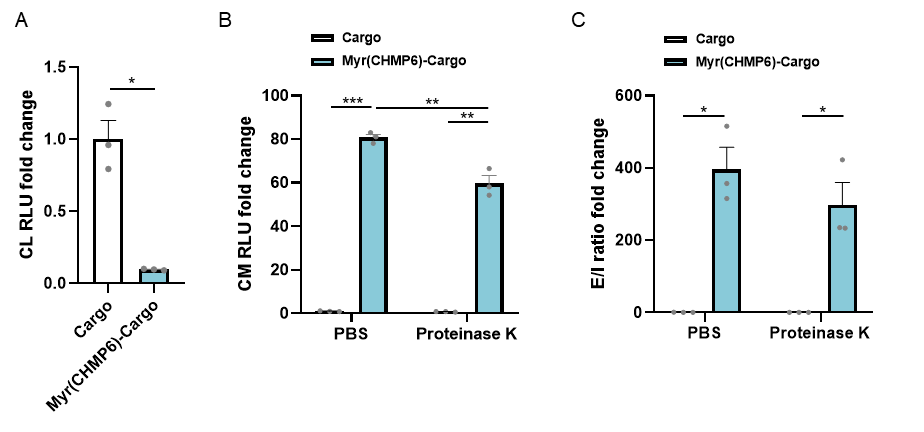


**Figure S3. NanoGlo luciferase assay on proteinase K-treated culture mediums (CM).** (A) Fold change values of cell lysate (CL) RLU. (B) Fold change values of CM RLU. (C) The E/I ratio was calculated by dividing Nluc activity in the culture medium by Nluc activity in cell lysates (CL) of EV-producing cells. E/I ratio fold change, fold change values of extracellular-to-intracellular RLU ratio. Bar graphs present fold change values compared to the first group as means ± standard error of the mean (SEM) (N ≥ 3). Statistical significance was assessed using unpaired two-tailed Student’s *t*-tests. **p* < 0.05, ***p* < 0.01, ****p* < 0.001.


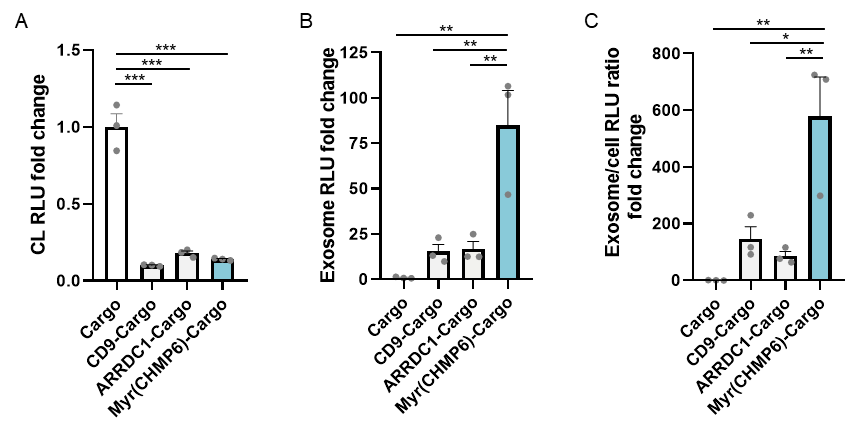


**Figure S4. NanoGlo luciferase assay on CD63+ exosomes.** (A) Fold change values of cell lysate (CL) RLU. (B) Fold change values of CD63+ exosome RLU. (C) Fold change values of RLU ratio of CD63+ exosomes vs. their producing cells. Data are presented as means ± SEM (N = 3). One-way ANOVA and Bonferroni correction were used for multiple comparisons to obtain adjusted *p*-values. **p* < 0.05, ***p* < 0.01, ****p* < 0.001.


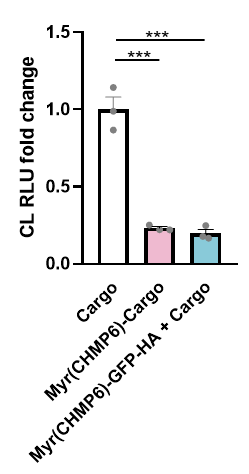


**Figure S5. Nluc activity of cell lysates (CL) corresponding to Figure 4A and 4B.** Bar graphs present fold change values compared to the first group as means ± standard error of the mean (SEM) (N = 3). One-way ANOVA and Bonferroni correction were used for multiple comparisons to obtain adjusted *p*-values. ****p* < 0.001.


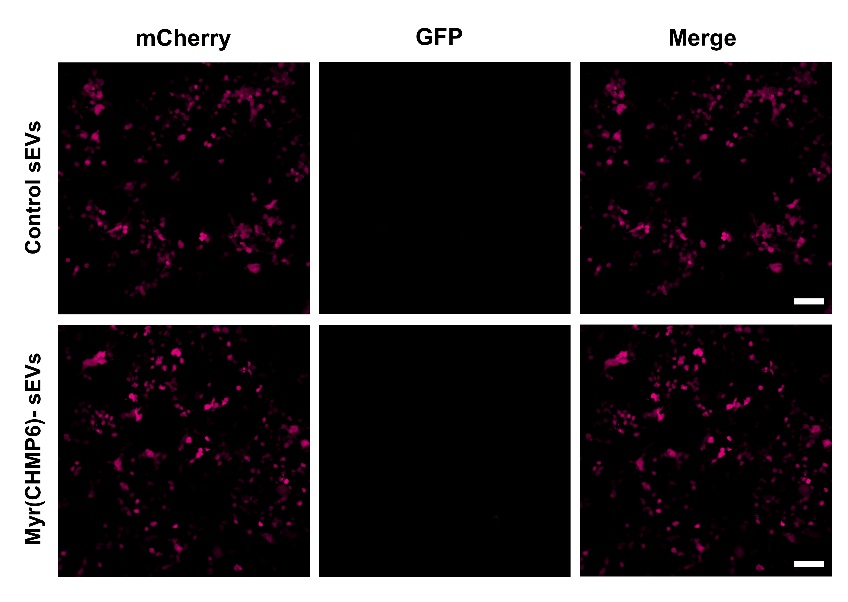


**Figure S6. Flouresence images of recipient cells related to Figure 5.** sEVs from donor cells that express Myr(CHMP6)-GFP-HA are termed Myr(CHMP6)-sEVs. sEVs from donor cells that express are termed control sEVs. LentiX-293T recipient cells were treated with sEVs loaded with a control cargo mCherry-Nluc-3×FLAG (rather than gene editing proteins), followed by transfection of the fluorescent reporter system. Scale bar represents 100 μm.


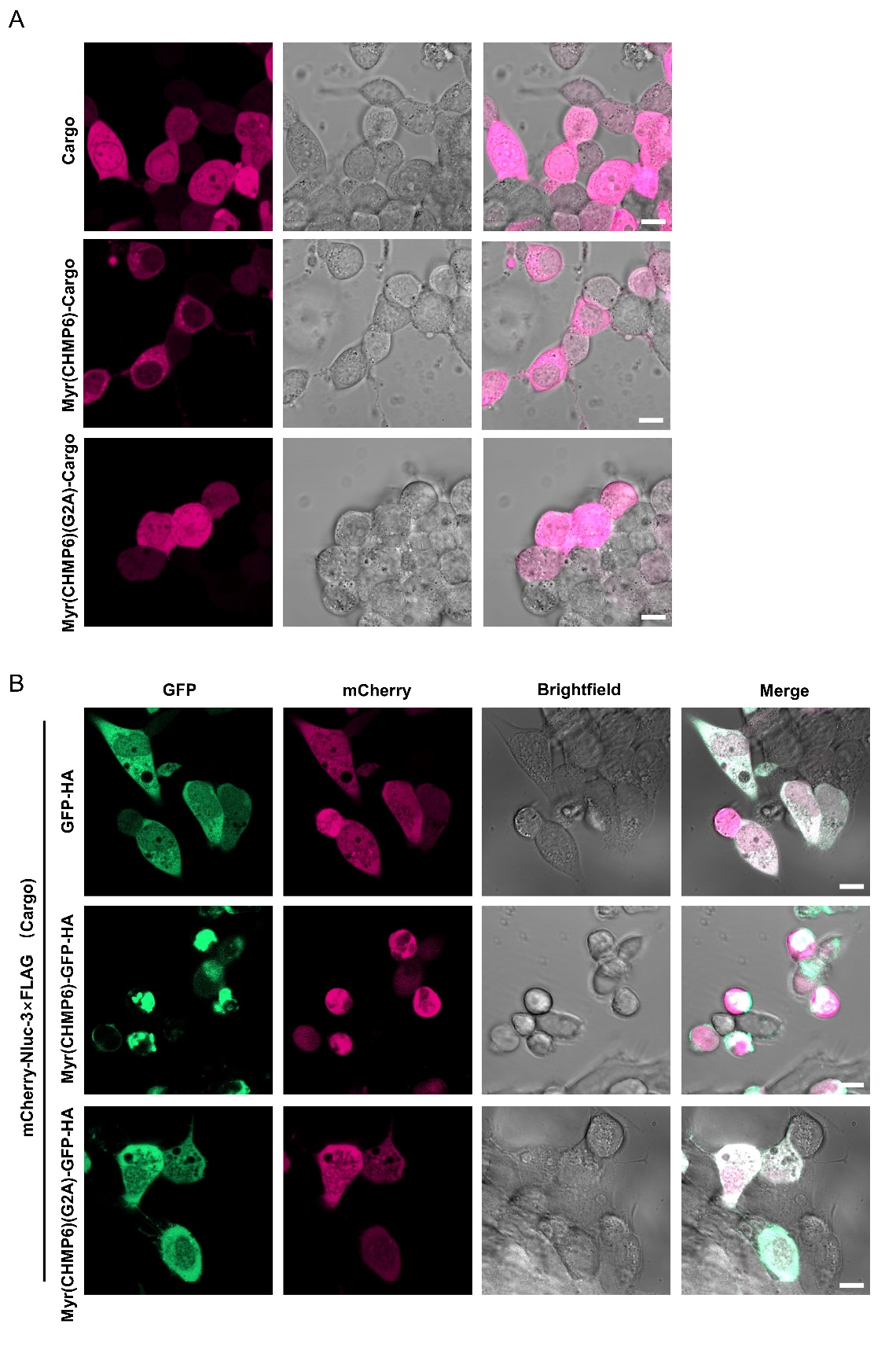


**Figure S7. Fluorescence and bright field images of Myr(CHMP6)(G2A) mutants and co-expressed cargoes.** (A) Images of Myr(CHMP6)(G2A)-Cargo. (B) Cargo co-expressed with GFP-HA, Myr(CHMP6)-GFP-HA, or the mutated Myr(CHMP6)(G2A)-GFP-HA. Scale bar represents 10 μm.


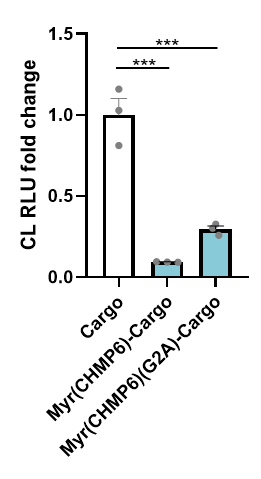


**Figure S8. Nluc activity of cell lysates (CL) corresponding to Figure 7E and 7F.** Bar graphs present fold change values compared to the first group as means ± standard error of the mean (SEM) (N = 3). One-way ANOVA and Bonferroni correction were used for multiple comparisons to obtain adjusted *p*-values. ****p* < 0.001.

Supplementary Tables

### Table S1. Primers

| **Name** | **Sence primer (5’-3’)** | **Antisence primer (5’-3’)** |
| --- | --- | --- |
| Nluc-3×FLAG | CGGCATGGACGAGCTGT | GTAATCCAGAGGTTGATTGTCGACTCATCCGCTCGAGCCGCCT |
| CD9 | AAGACACCGACTCTAGAGGCCGCCACCATGCCGGTCAAAGGAGGC | GCTTCCTCCTCCTCCGCTTCCACCTCCTCCAGCGCTGACCATCTCGCGGTTCCTG |
| ARRDC1 | AAGACACCGACTCTAGAGGCCGCCACCATGGGGCGAGTGCAGCTC | CGCTTCCACCTCCTCCGGATCCGCTCTCAGGGGTCAGGCTG |
| Ubi | AGAAGACACCGACTCTAGAGCCACCATGCAGATTTTCGTGA | GGAACCGCCGCCCCCAGAACCACCACGAAGTCTCAACACAAGA |
| SHH | GGTGGAGGCGGCGGATCCGGAGGCTGCTTCCCGGGCT | GTAATCCAGAGGTTGATTGTCGACTCAGCTGGACTTGACCGCCATG |
| Myr(TCRV) | GACCTCCATAGAAGACACCGACTCTAGAGCCGCCACCATGGGCA | GCTACCACCGCCGCCACCGGTACTACCGCCACCT |
| Myr(Gag) | TAGTGAACCGTCAGATCGCCT | CTACCACCGCCGCCACCGGTACTACCGCCTCCCCCGCTTAATACTGACGCT |
| Myr(CHMP3) | AGACACCGACTCTAGAGGCCGCCACCATGGGGCTGTTTGGAAAGACCCAGGAGAAGCC | CTACCACCGCCGCCACCGGTACTACCGCCTTTGGGCGGCTTCTCCTGGGTCTTTCCA |
| Myr(CHMP6) | AGACACCGACTCTAGAGGCCGCCACCATGGGTAACCTGTTCGGCCGCAAGAAGCAGA | CTACCACCGCCGCCACCGGTACTACCGCCGCGGCTCTGCTTCTTGCGGCCGAACA |
| Prenyl(RAB5A) | ACGATAAGGGTGGAGGCGGCTCGAGCCAACCAACCAGGAATCAGTGTTGT | AATCCAGAGGTTGATTGTCGACTTAGTTACTACAACACTGATTCCTGGTTGGT |
| Prenyl(RAB7A) | TGGAGGCGGCTCGAGCAAACTGGACAAGAATGACCGGGCCAAGGCCTCGGCAGAAAGCT | AATCCAGAGGTTGATTGTCGACTCAGCAACTGCAGCTTTCTGCCGAGGCCTT |
| Prenyl(RAB11) | GTGGAGGCGGCTCGAGCCCAACCACTGAAAACAAGCCAAAGGTGCAGTGCTGTCAGA | AATCCAGAGGTTGATTGTCGACTTAGATGTTCTGACAGCACTGCACCTTTGGCT |
| HA | TTCTGCTAGGATCAATGTGGGAGGAGGTGGCTCGAGCGGATACCCATACG | ATCCAGAGGTTGATTGTCGACCTAAGCGTAATCTGGAACATCGTATGGG |
| GFP-1 | GACACCGACTCTAGAGGATCCGCCGCCACCATGCCCG | CCCACATTGATCCTAGCAGAAGCACAG |
| GFP-2 | AGGAGGCGGTAGCACTAGTATGCCCGCCATGAAGATCG | CCCACATTGATCCTAGCAGAAGCACAG |
| Cre-3×FLAG | ATAGAAGACACCGACTCTAGAGGCCGCCACCATGCCCAAGAAGAAGAGGAAGGTGT | GTTGATTGTCGACTCAGGATCCGCCGCCTCCACCCTTATCGTC |
| CHMP6(G2A)-1 | AAGACACCGACTCTAGAGGCCGCCACCATGGCTAAC | CTCCGCCACTAGACTTGTACAGCTCGT |
| CHMP6(G2A)-2 | AAGACACCGACTCTAGAGGCCGCCACCATGGCTAAC | ATCCAGAGGTTGATTGTCGACCTAAGCGTAATCTGGAACATCGTATGGG |

### Table S2. Primary antibodies

| **Antibodies** | **Clone** | **Source** | **Identifier/Cat No.** | **Dilution** |
| --- | --- | --- | --- | --- |
| HA tag | Polyclonal | Proteintech | 51064-1-AP | 1:4000 |
| DYKDDDDK tag | Polyclonal | Proteintech | 20543-1-AP | 1:2000 |
| Calnexin | 2A2C6 | Proteintech | 66903-1-Ig | 1:10000 |
| CD81 | 1G2C6 | Proteintech | 66866-1-Ig | 1:2000 |
| Alix | Polyclonal | Proteintech | 12422-1-AP | 1:2000 |
| GAPDH | 1E6D9 | Proteintech | 60004-1-Ig | 1:20000 |
