# Supplementary material for: A peptide derived from the N-terminus of charged multivesicular body protein 6 (CHMP6) promotes the secretion of gene editing proteins via small extracellular vesicle production
Source: Bioengineered. 2022 Feb 21;13(3):4702–16. doi: 10.1080/21655979.2022.2030571 (PMC8973635; doi:10.1080/21655979.2022.2030571)
Supplement: Supplemental Material [file KBIE_A_2030571_SM1069.zip › supplementary/Supplementary Tables.docx]

Supplementary Tables

### Table S1. Primers

| **Name** | **Sence primer (5’-3’)** | **Antisence primer (5’-3’)** |
| --- | --- | --- |
| Nluc-3×FLAG | CGGCATGGACGAGCTGT | GTAATCCAGAGGTTGATTGTCGACTCATCCGCTCGAGCCGCCT |
| CD9 | AAGACACCGACTCTAGAGGCCGCCACCATGCCGGTCAAAGGAGGC | GCTTCCTCCTCCTCCGCTTCCACCTCCTCCAGCGCTGACCATCTCGCGGTTCCTG |
| ARRDC1 | AAGACACCGACTCTAGAGGCCGCCACCATGGGGCGAGTGCAGCTC | CGCTTCCACCTCCTCCGGATCCGCTCTCAGGGGTCAGGCTG |
| Ubi | AGAAGACACCGACTCTAGAGCCACCATGCAGATTTTCGTGA | GGAACCGCCGCCCCCAGAACCACCACGAAGTCTCAACACAAGA |
| SHH | GGTGGAGGCGGCGGATCCGGAGGCTGCTTCCCGGGCT | GTAATCCAGAGGTTGATTGTCGACTCAGCTGGACTTGACCGCCATG |
| Myr(TCRV) | GACCTCCATAGAAGACACCGACTCTAGAGCCGCCACCATGGGCA | GCTACCACCGCCGCCACCGGTACTACCGCCACCT |
| Myr(Gag) | TAGTGAACCGTCAGATCGCCT | CTACCACCGCCGCCACCGGTACTACCGCCTCCCCCGCTTAATACTGACGCT |
| Myr(CHMP3) | AGACACCGACTCTAGAGGCCGCCACCATGGGGCTGTTTGGAAAGACCCAGGAGAAGCC | CTACCACCGCCGCCACCGGTACTACCGCCTTTGGGCGGCTTCTCCTGGGTCTTTCCA |
| Myr(CHMP6) | AGACACCGACTCTAGAGGCCGCCACCATGGGTAACCTGTTCGGCCGCAAGAAGCAGA | CTACCACCGCCGCCACCGGTACTACCGCCGCGGCTCTGCTTCTTGCGGCCGAACA |
| Prenyl(RAB5A) | ACGATAAGGGTGGAGGCGGCTCGAGCCAACCAACCAGGAATCAGTGTTGT | AATCCAGAGGTTGATTGTCGACTTAGTTACTACAACACTGATTCCTGGTTGGT |
| Prenyl(RAB7A) | TGGAGGCGGCTCGAGCAAACTGGACAAGAATGACCGGGCCAAGGCCTCGGCAGAAAGCT | AATCCAGAGGTTGATTGTCGACTCAGCAACTGCAGCTTTCTGCCGAGGCCTT |
| Prenyl(RAB11) | GTGGAGGCGGCTCGAGCCCAACCACTGAAAACAAGCCAAAGGTGCAGTGCTGTCAGA | AATCCAGAGGTTGATTGTCGACTTAGATGTTCTGACAGCACTGCACCTTTGGCT |
| HA | TTCTGCTAGGATCAATGTGGGAGGAGGTGGCTCGAGCGGATACCCATACG | ATCCAGAGGTTGATTGTCGACCTAAGCGTAATCTGGAACATCGTATGGG |
| GFP-1 | GACACCGACTCTAGAGGATCCGCCGCCACCATGCCCG | CCCACATTGATCCTAGCAGAAGCACAG |
| GFP-2 | AGGAGGCGGTAGCACTAGTATGCCCGCCATGAAGATCG | CCCACATTGATCCTAGCAGAAGCACAG |
| Cre-3×FLAG | ATAGAAGACACCGACTCTAGAGGCCGCCACCATGCCCAAGAAGAAGAGGAAGGTGT | GTTGATTGTCGACTCAGGATCCGCCGCCTCCACCCTTATCGTC |
| CHMP6(G2A)-1 | AAGACACCGACTCTAGAGGCCGCCACCATGGCTAAC | CTCCGCCACTAGACTTGTACAGCTCGT |
| CHMP6(G2A)-2 | AAGACACCGACTCTAGAGGCCGCCACCATGGCTAAC | ATCCAGAGGTTGATTGTCGACCTAAGCGTAATCTGGAACATCGTATGGG |

### Table S2. Primary antibodies

| **Antibodies** | **Clone** | **Source** | **Identifier/Cat No.** | **Dilution** |
| --- | --- | --- | --- | --- |
| HA tag | Polyclonal | Proteintech | 51064-1-AP | 1:4000 |
| DYKDDDDK tag | Polyclonal | Proteintech | 20543-1-AP | 1:2000 |
| Calnexin | 2A2C6 | Proteintech | 66903-1-Ig | 1:10000 |
| CD81 | 1G2C6 | Proteintech | 66866-1-Ig | 1:2000 |
| Alix | Polyclonal | Proteintech | 12422-1-AP | 1:2000 |
| GAPDH | 1E6D9 | Proteintech | 60004-1-Ig | 1:20000 |
