# Supplementary figures and images for: A peptide derived from the N-terminus of charged multivesicular body protein 6 (CHMP6) promotes the secretion of gene editing proteins via small extracellular vesicle production
Source: Bioengineered. 2022 Feb 21;13(3):4702–16. doi: 10.1080/21655979.2022.2030571 (PMC8973635; doi:10.1080/21655979.2022.2030571)

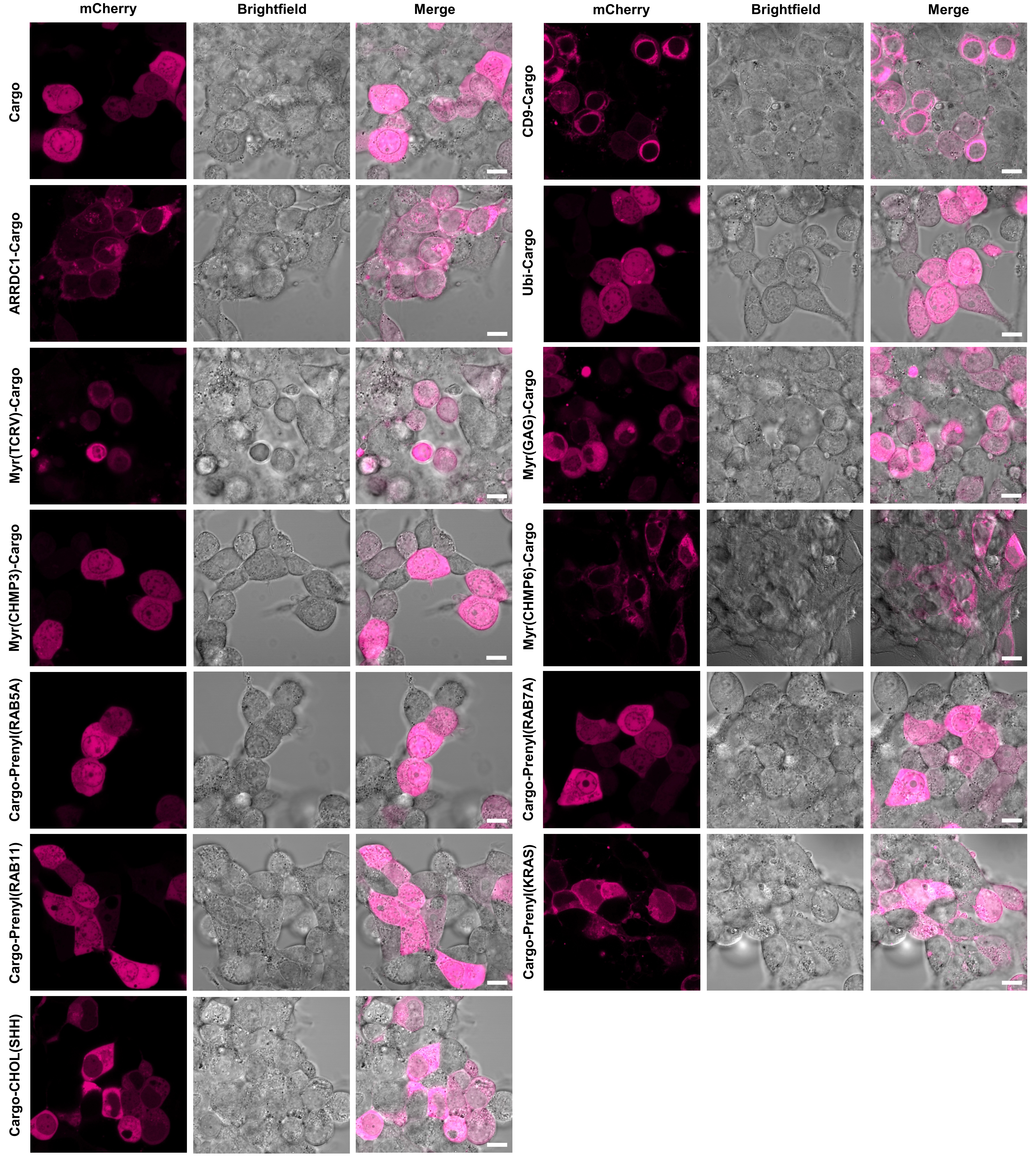

Supplement: Supplemental Material [file KBIE_A_2030571_SM1069.zip › supplementary/FigS1.jpg]

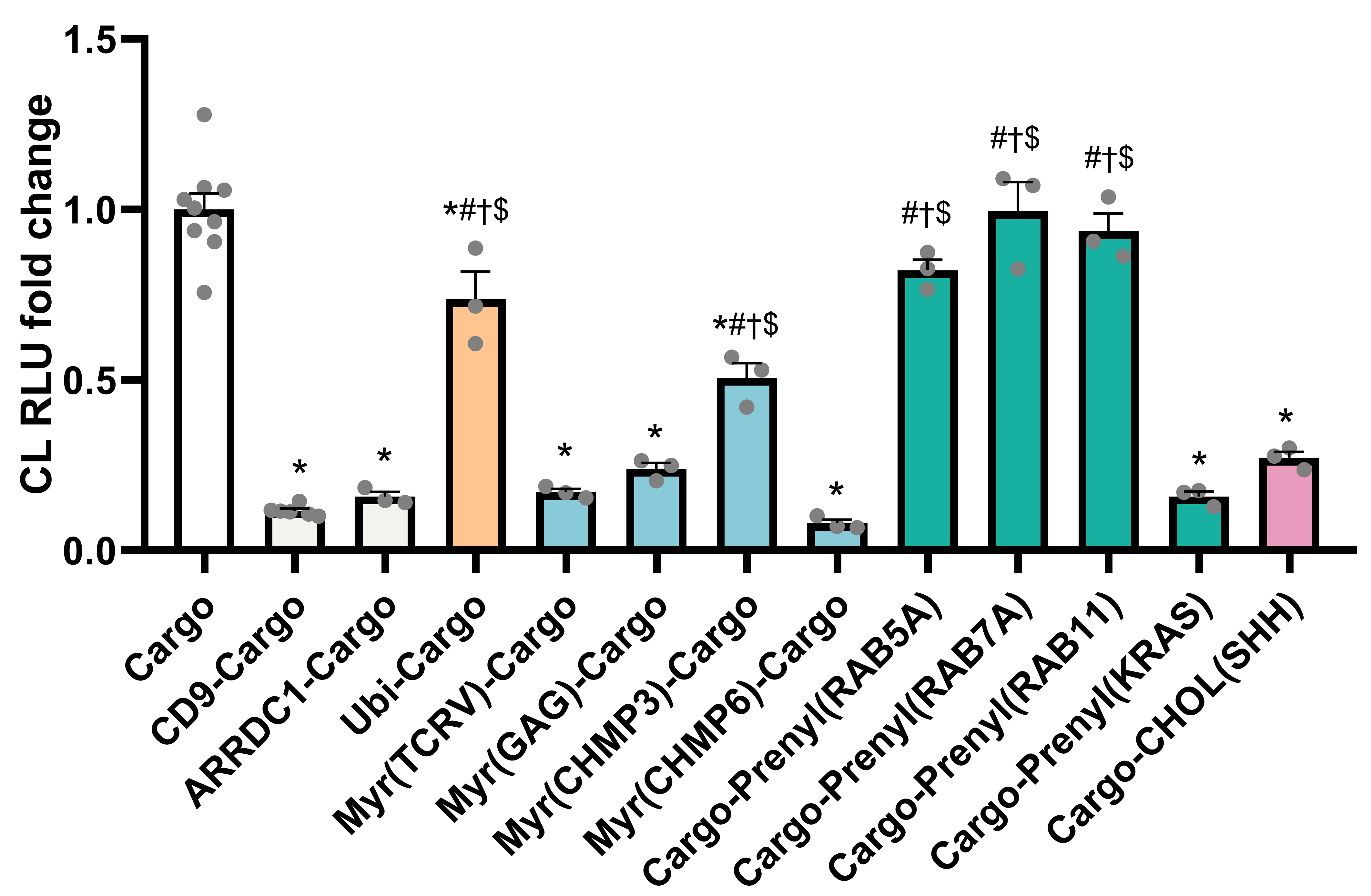

Supplement: Supplemental Material [file KBIE_A_2030571_SM1069.zip › supplementary/FigS2.TIF]

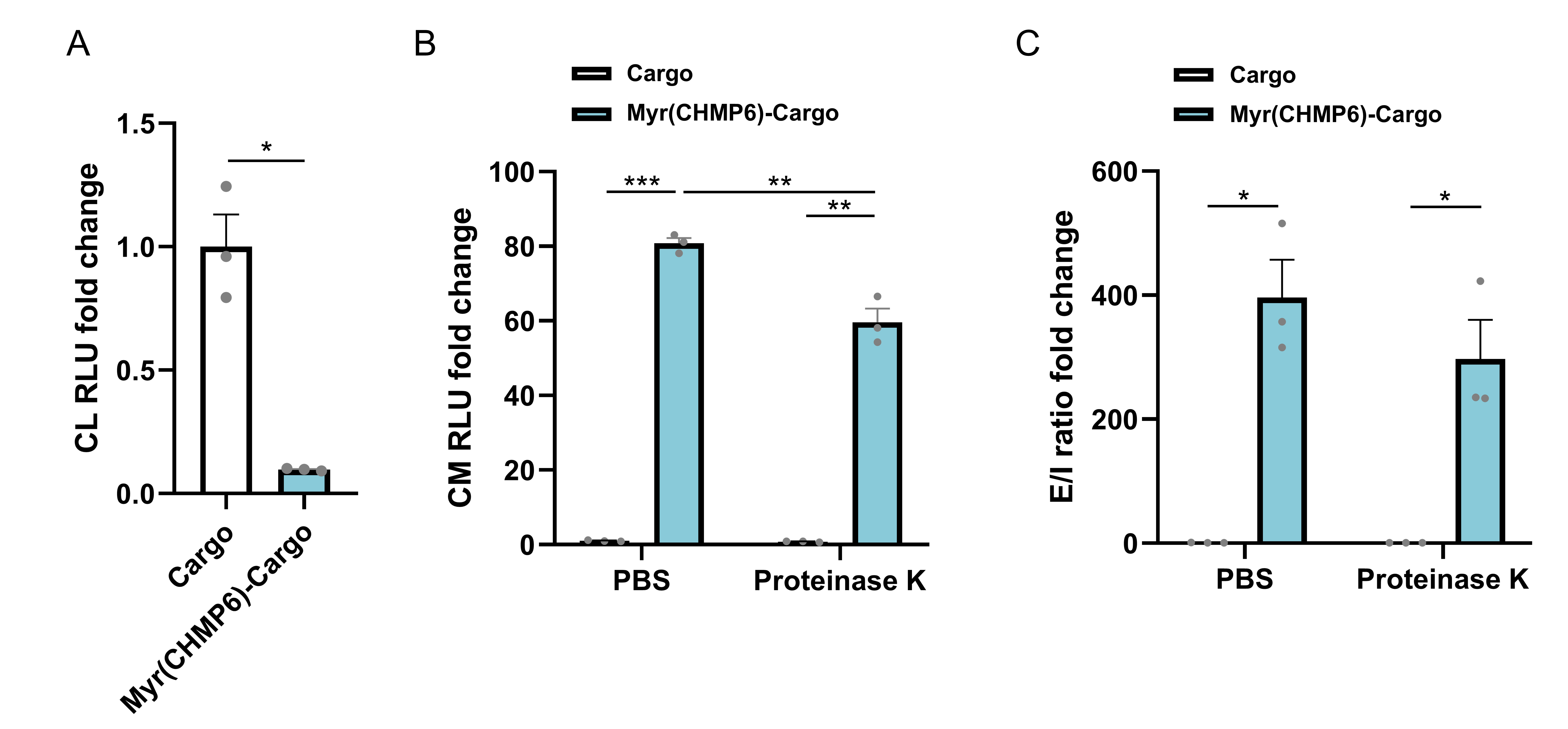

Supplement: Supplemental Material [file KBIE_A_2030571_SM1069.zip › supplementary/FigS3.TIF]

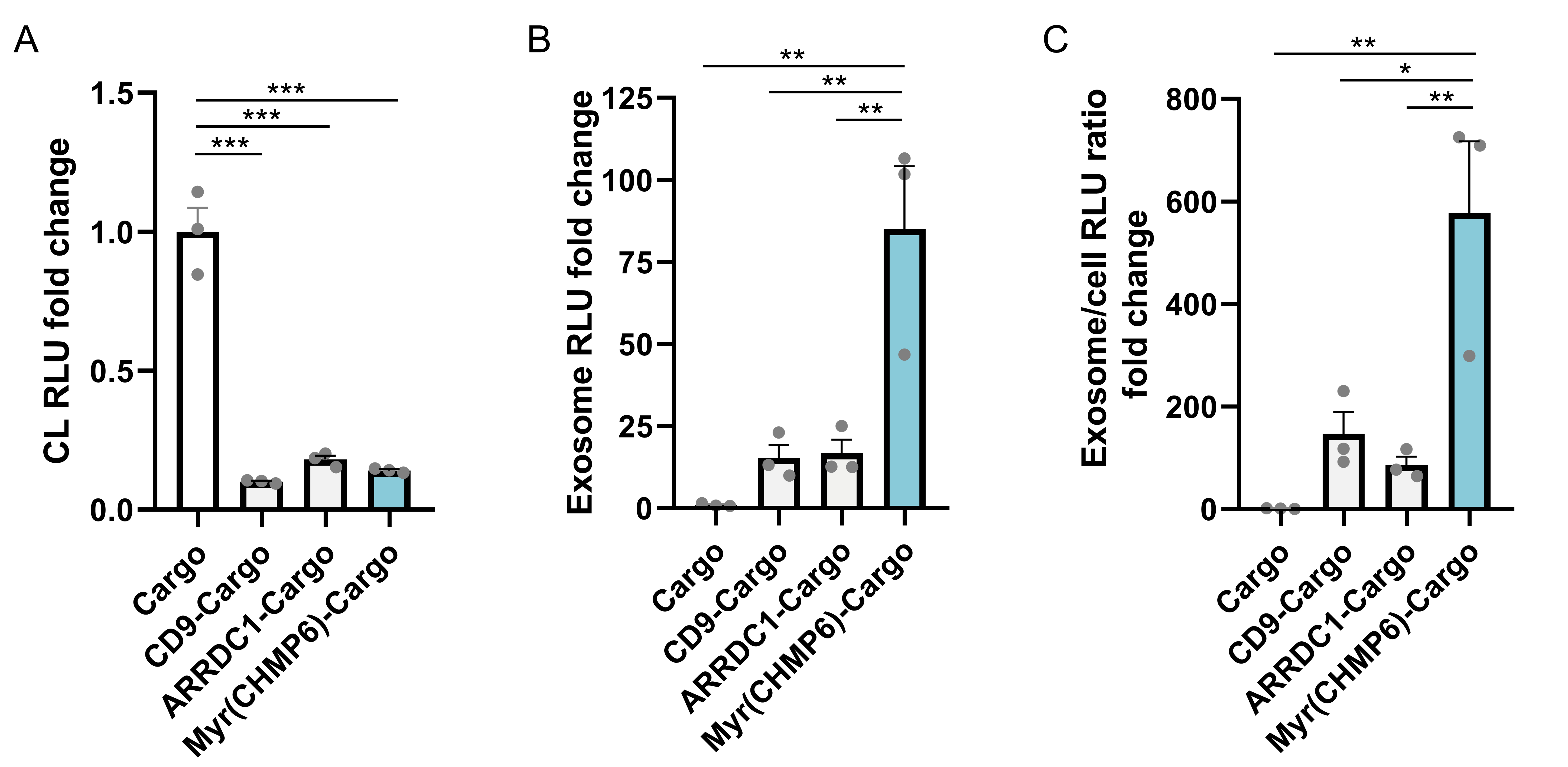

Supplement: Supplemental Material [file KBIE_A_2030571_SM1069.zip › supplementary/FigS4.TIF]

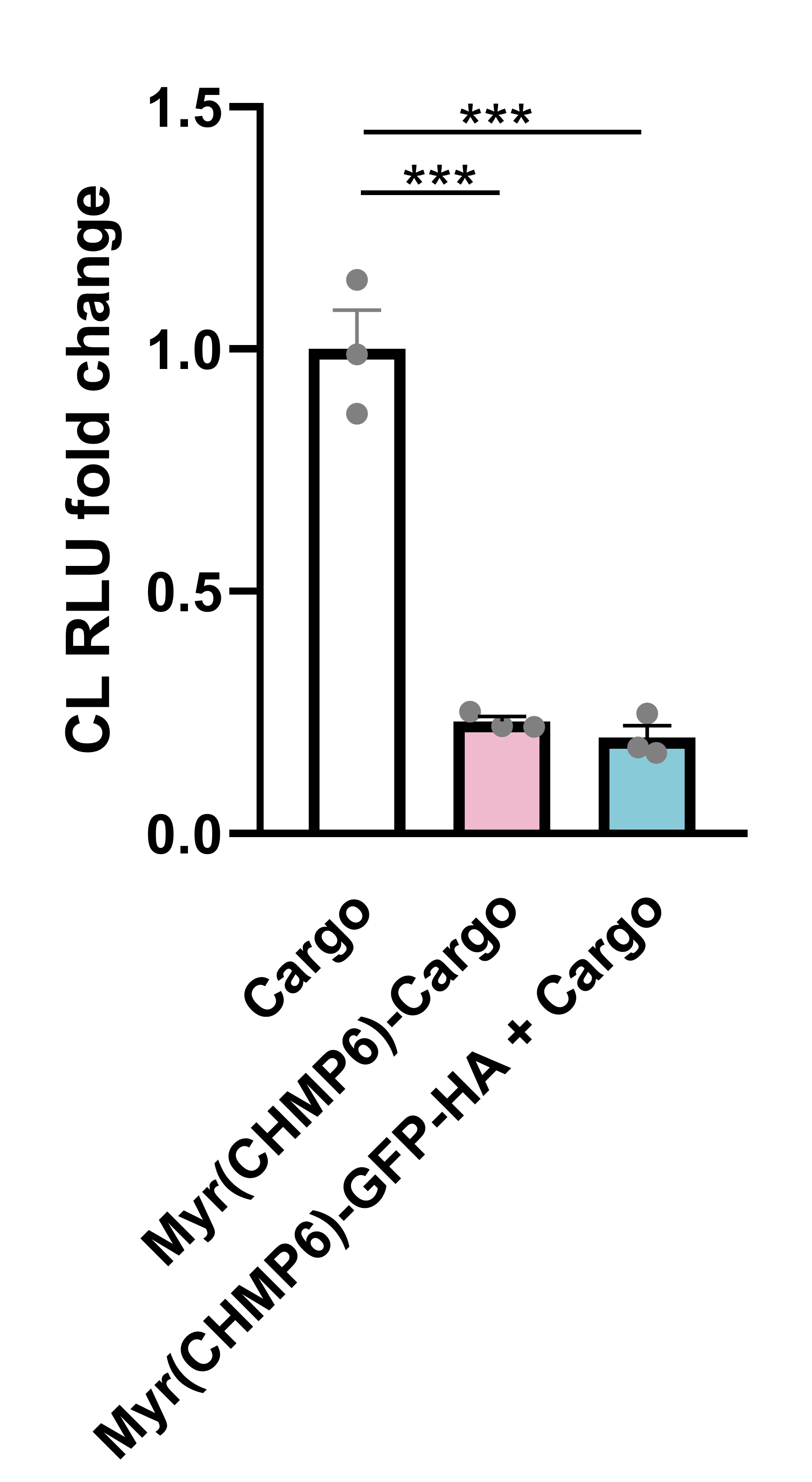

Supplement: Supplemental Material [file KBIE_A_2030571_SM1069.zip › supplementary/FigS5.TIF]

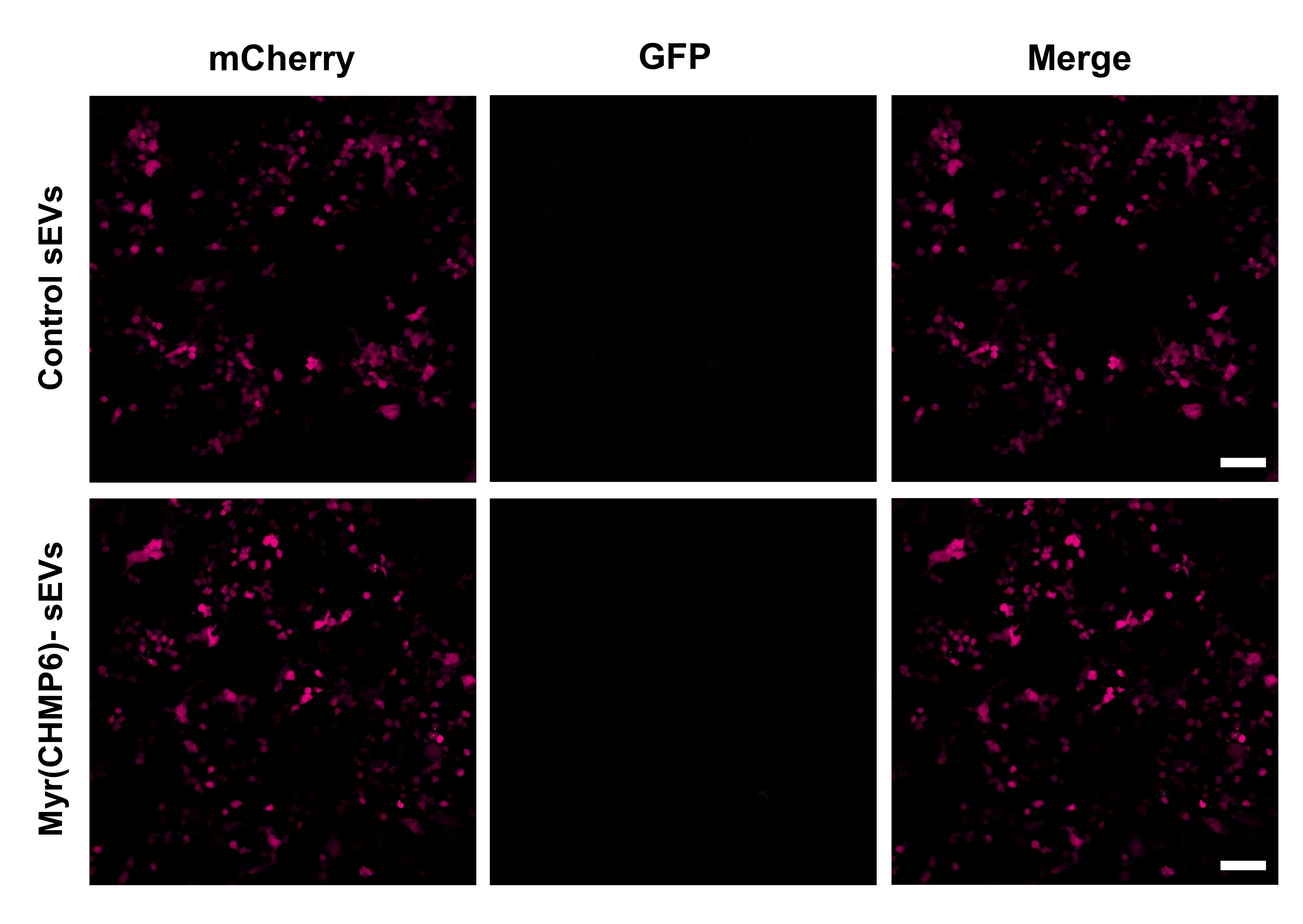

Supplement: Supplemental Material [file KBIE_A_2030571_SM1069.zip › supplementary/FigS6.jpg]

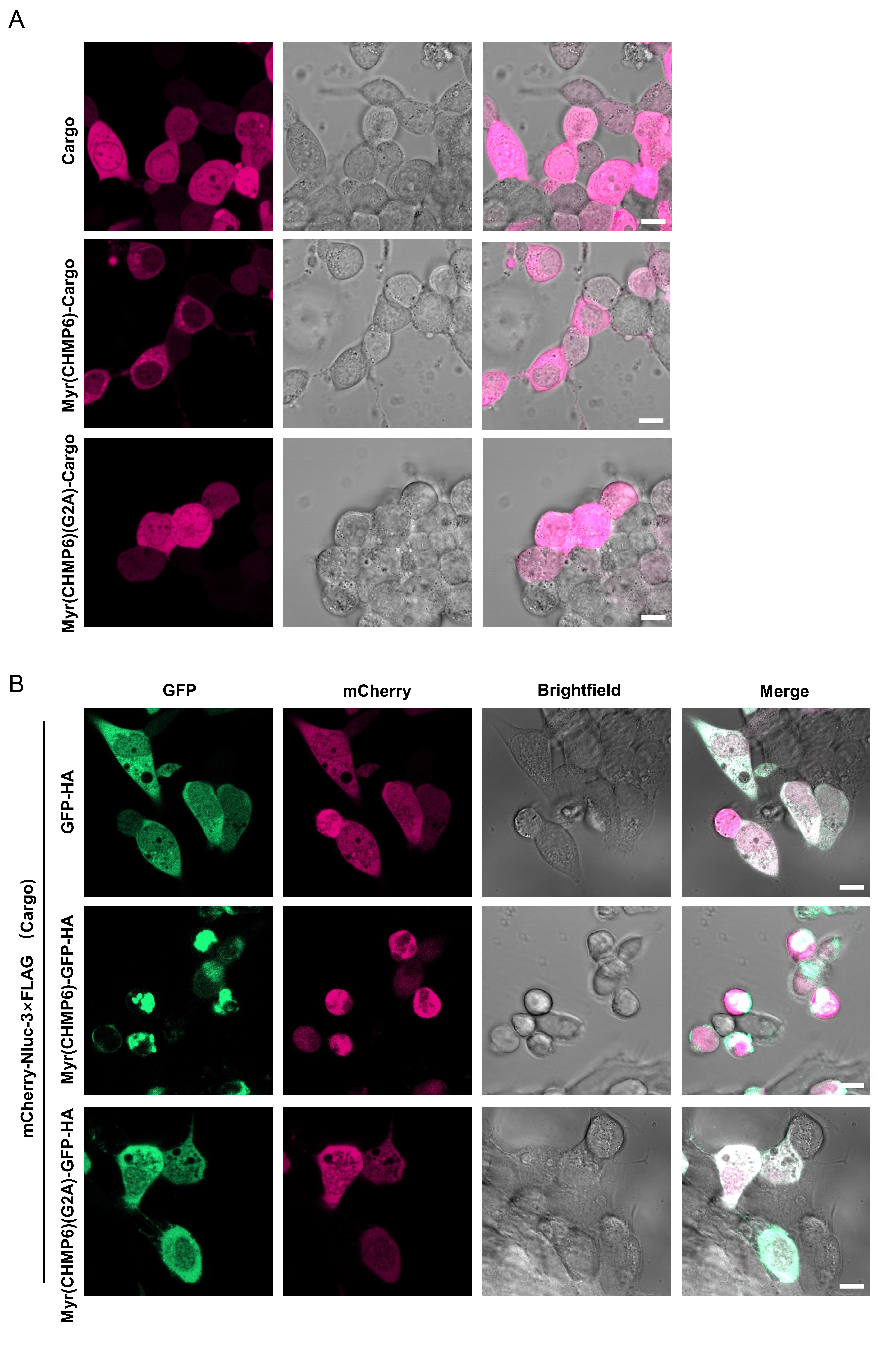

Supplement: Supplemental Material [file KBIE_A_2030571_SM1069.zip › supplementary/FigS7.jpg]

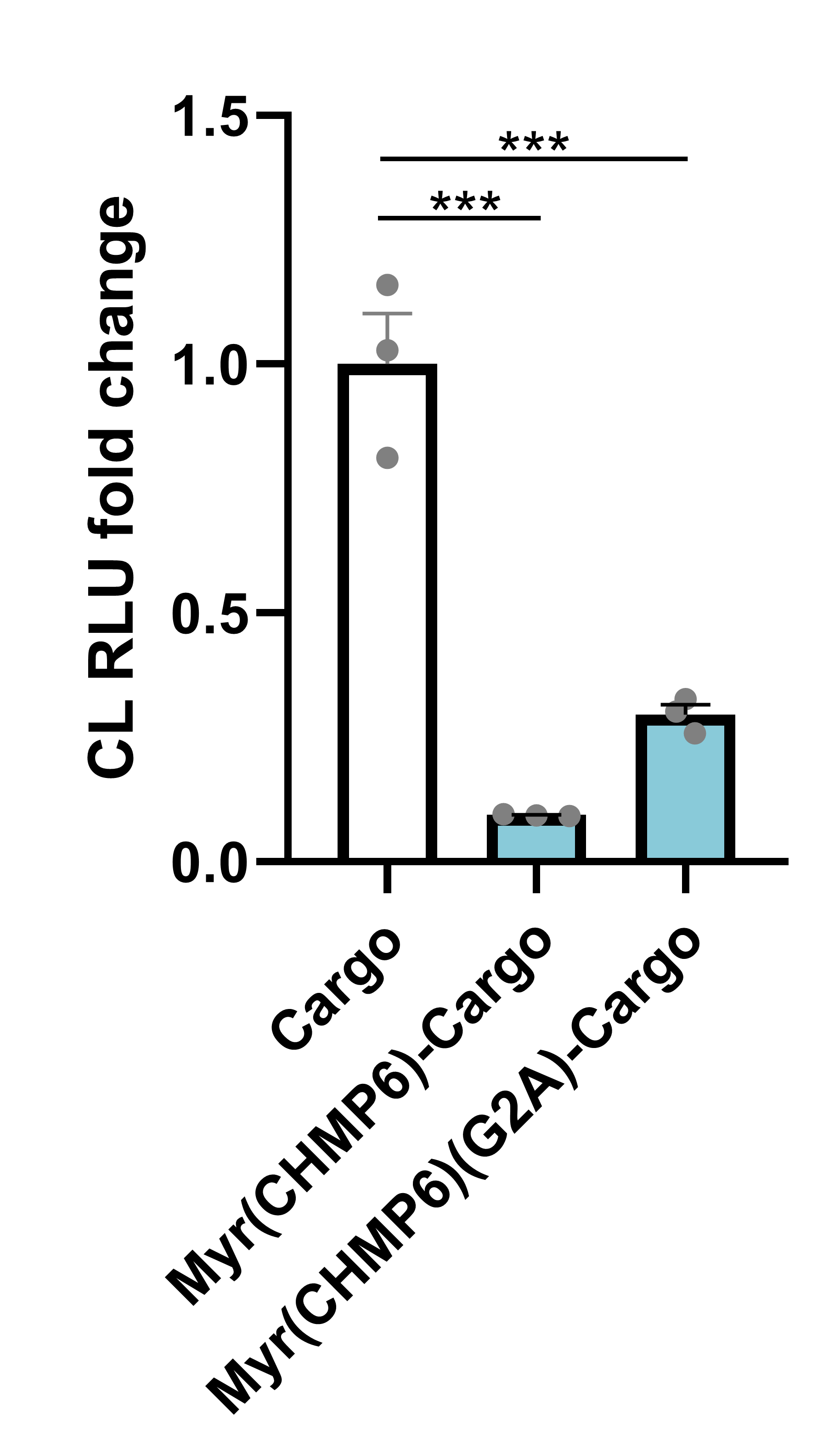

Supplement: Supplemental Material [file KBIE_A_2030571_SM1069.zip › supplementary/FigS8.TIF]
